# Disorder and the Neural Representation of Complex Odors

**DOI:** 10.3389/fncom.2022.917786

**Published:** 2022-08-08

**Authors:** Kamesh Krishnamurthy, Ann M. Hermundstad, Thierry Mora, Aleksandra M. Walczak, Vijay Balasubramanian

**Affiliations:** ^1^Joseph Henry Laboratories of Physics and Princeton Neuroscience Institute, Princeton University, Princeton, NJ, United States; ^2^Janelia Research Campus, Howard Hughes Medical Institute, Ashburn, VA, United States; ^3^Laboratoire de Physique Statistique, UMR8550, CNRS, UPMC and École Normale Supérieure, Paris, France; ^4^Laboratoire de Physique Théorique, UMR8549m CNRS, UPMC and École Normale Supérieure, Paris, France; ^5^David Rittenhouse and Richards Laboratories, University of Pennsylvania, Philadelphia, PA, United States

**Keywords:** olfaction, efficient coding, sensory neuroscience, Piriform Cortex, olfactory bulb, olfactory receptor

## Abstract

Animals smelling in the real world use a small number of receptors to sense a vast number of natural molecular mixtures, and proceed to learn arbitrary associations between odors and valences. Here, we propose how the architecture of olfactory circuits leverages disorder, diffuse sensing and redundancy in representation to meet these immense complementary challenges. First, the diffuse and disordered binding of receptors to many molecules compresses a vast but sparsely-structured odor space into a small receptor space, yielding an odor code that preserves similarity in a precise sense. Introducing any order/structure in the sensing degrades similarity preservation. Next, lateral interactions further reduce the correlation present in the low-dimensional receptor code. Finally, expansive disordered projections from the periphery to the central brain reconfigure the densely packed information into a high-dimensional representation, which contains multiple redundant subsets from which downstream neurons can learn flexible associations and valences. Moreover, introducing any order in the expansive projections degrades the ability to recall the learned associations in the presence of noise. We test our theory empirically using data from *Drosophila*. Our theory suggests that the neural processing of sparse but high-dimensional olfactory information differs from the other senses in its fundamental use of disorder.

## 1. Introduction

Animals sense and respond to volatile molecules that carry messages from and about the world. Some kinds of olfactory behaviors require sensing of particular molecules such as pheromones. These molecules and the receptors that bind to them have likely co-evolved over long periods of time to ensure precise and specific binding. However, to be useful in a diverse and changing world, the olfactory system should be prepared to sense and process any volatile molecule. There are a very large number of such monomolecular odorants (Dunkel et al., 2009; Touhara and Vosshall, 2009; Mayhew et al., 2020), far more than the number of receptor types available to bind these odorants. Humans, flies and mice, for instance, have just about 300, 500, and 1,000 functional olfactory receptor types, respectively (Vosshall et al., 2000; Zozulya et al., 2001; Zhang and Firestein, 2002). Yet, animals may be able to discriminate between orders of magnitude more odors than the number of receptor types (a high estimate is given in Bushdid et al., 2014, but see Gerkin and Castro, 2015).

At an abstract level, the early stage of the olfactory system faces the immense challenge of embedding a very high-dimensional input space (the space of odor molecules) into a low-dimensional space of sensors (the response space of olfactory receptors). The distributed and combinatorial nature of receptor responses in part tackles this problem (Malnic et al., 1999; Araneda et al., 2000; Laurent et al., 2001; Stopfer et al., 2003; Kay and Stopfer, 2006; Bazhenov and Stopfer, 2009; Saito et al., 2009; Stevens, 2015; Zhang and Sharpee, 2016). This embedding must also preserve similarity between different odors well enough to permit the judgements of sameness and difference that are crucial for behavior. Furthermore, experiments (Choi et al., 2011) suggest that this odor representation is reorganized in higher brain regions to be enormously flexible, allowing learning of nearly arbitrary associations between valences and different groups of odors.

**Figure 1:**
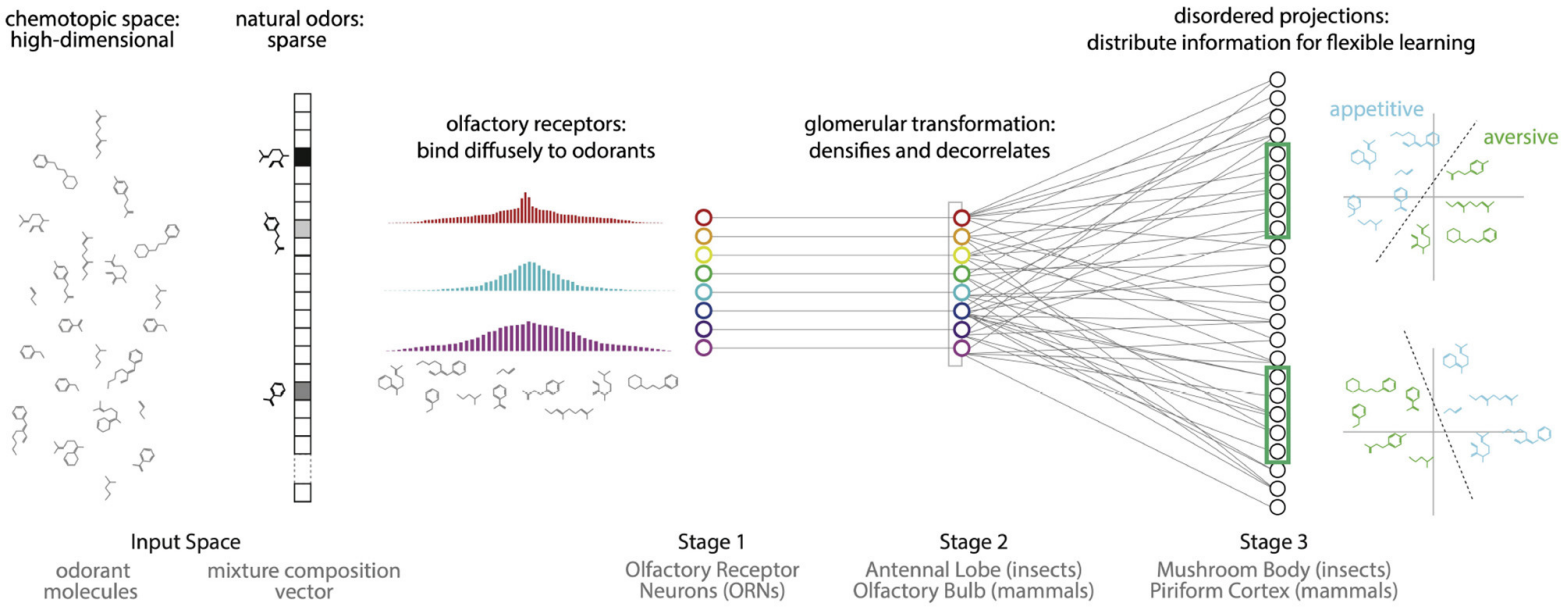
Proposal: The olfactory system uses two kinds of disorder to first compress odor information into the responses of a small number of receptors, and then reconfigure this information to enable flexible associations between odors and valences. (i) Natural odors are high dimensional but sparse: they contain a tiny fraction of all possible monomolecular odorants. (ii) Olfactory receptors diffusely bind to a broad range of odorants, producing a compact representation of odor information that enables accurate decoding. (iii) The Antennal Lobe/Olfactory Bulb decorrelates this representation. (iv) Disordered projections from the Antennal Lobe/Olfactory Bulb to the Mushroom Body/Piriform Cortex, followed by non-linearities, create a sparse and distributed representation of odors that facilitates flexible learning of odor categories from small and arbitrarily-chosen subsets of neurons.

Here, using an end-to-end integrated model (Figure 1), we provide empirical evidence that the olfactory system powerfully exploits physiological and structural *disorder*—or lack of structure—at different stages of processing to meet these two complementary challenges: (*i*) compression of a vast but sparsely-structured odor space into a similarity preserving receptor code, and (*ii*) reorganization of the receptor code into a high-dimensional representation, which allows flexible learning from redundant subsets of neurons. The benefits of such expansive projections for learning have been studied before from the perspective of capacity, sparsity and robustness to noise (Haberly, 2001; Luo et al., 2010; Babadi and Sompolinsky, 2014; Dasgupta et al., 2017; Litwin-Kumar et al., 2017). Here our focus is on the effect of disorder, and on flexible learning of associations from redundant subsets. We also focus on the time-averaged properties of the combinatorial receptor responses, thus omitting receptor and circuit dynamics, which can be relevant in some olfactory phenomena (Rabinovich et al., 2000; Laurent et al., 2001; Laurent, 2002; Stopfer et al., 2003; Brown et al., 2005; Mazor and Laurent, 2005; Turner et al., 2008; Raman et al., 2010; Nagel and Wilson, 2011; Gupta and Stopfer, 2014; Sanda et al., 2016) (but also c.f. Stevens, 2015; Zhang and Sharpee, 2016; Dasgupta et al., 2017; Grabska-Barwińska et al., 2017; Hiratani and Latham, 2020).

To perform effectively within its design constraints, a sensory system must exploit structure in the environment. For example, the symmetries and statistics of natural images dictate an efficient decomposition into edges (Olshausen and Field, 1996), likely explaining why simple cells in the visual cortex respond preferentially to oriented lines and why complex cell responses are invariant to translations within the receptive field (Hubel and Wiesel, 1962; Riesenhuber and Poggio, 2000). We noted that a salient feature of natural odors is that they typically contain only a tiny fraction of the possible volatile molecular species (Krishnamurthy et al., 2014). For example, food odors are typically composed of 3–40 molecules (Yu et al., 2015). Natural odors are thus *sparse* in the high-dimensional space of odorant molecules. Surprising results from the mathematical literature on random projections (Candès et al., 2006; Donoho, 2006; Baraniuk et al., 2010) show that there is an efficient solution for storing signals of this nature: sparse, high-dimensional input signals can be encoded—in a manner preserving similarity—by a compact set of sensors through diffuse and disordered measurements of the input space. For example, this sort of compression can be achieved if each sensor response contains randomly weighted contributions from every dimension of the input space. Importantly, this diffuse sensing need not be tuned to the specific structure of the input signal—i.e., in this manner, it can be non-adaptive. This sensing scheme contrasts with the expectation for many sensory modalities in that a lack of structure/symmetry in the responses is required to efficiently capture the sparse structure of the inputs. We propose that the olfactory system employs such a diffuse sensing strategy in order to exploit the sparse structure of natural odor space, and to produce compact representations of odors (Figure 1).

Ultimately, odor representations must support associations between odors and valence, and experimental evidence suggests that animals can learn such associations both flexibly and reversibly (Choi et al., 2011). However, the compact representations achieved by diffuse sensing make such learning difficult. This is fundamentally an issue of the dimensionality of the representation, and it is well-known that increasing the dimensionality improves the capacity for learning (e.g., Cover, 1965; Babadi and Sompolinsky, 2014). Here, we show that the disorder in the expansive projections to cortex is further beneficial for flexible learning from redundant subsets of the high-dimensional representation.

We use an integrated end-to-end model with data from *Drosophila* to provide evidence for our proposal. We show that the diffuse responses of olfactory receptor neurons provide a compact representation of odor information while preserving similarity. Introducing structure in the responses degrades similarity preservation. We then show that the non-linear transformation in the second stage of olfactory processing (Antennal Lobe in insects; Olfactory Bulb in mammals), followed by the apparently disordered, expansive projection to the third stage of olfactory processing (Mushroom Body in insects; Piriform Cortex in mammals) creates a high-dimensional representation containing redundant copies of the information for flexible learning. Moreover, we show that introducing any structure in the expansive projections degrades the ability to recall the learned associations in the presence of noise.

## 2. Results

### 2.1. Olfactory Receptor Neurons Use Disorder to Encode Natural Odors: Decoding Error Analysis

Volatile molecules are sensed when they bind to olfactory receptors, each encoded by a separate gene (Buck and Axel, 1991). For example, in mice, almost 5% of the genome is devoted to encoding about 1, 000 receptor types. Even a relatively small number of olfactory receptors could in principle encode a vast number of odors because of the diffuse sensing of moelcules by ORNS (insect: Hallem and Carlson, 2006; Carey et al., 2010; mammal: Saito et al., 2009), and the consequent combinatorial nature of the odor code (Malnic et al., 1999; Stopfer et al., 2003; Stevens, 2015; Zhang and Sharpee, 2016). Indeed, the number of patterns of receptor response and silence increases exponentially with the number of receptors; *N* receptors have 2^*N*^ response/silence patterns so that just 50 receptors in Drosophila are capable of encoding over 10^15^ odors. However, a good odor code should also be structured to support judgements of similarity and difference that are essential to animal behavior. More formally, the code should preserve an appropriate notion of distance between odors. How could a combinatorial olfactory system where odorants bind diffusely to many receptors, and receptors bind to many odorants, preserve such distances?

Well-organized sensory systems perform their jobs well by adapting to structure in the environment. A key structure present in the olfactory environment is sparsity—natural odors typically contain a tiny fraction of the possible volatile molecules (Yu et al., 2015). More technically, the representation of a natural odor in terms of its molecular concentration vector will have very few non-vanishing components. Suppose there are *N* types of volatile molecules, and any given natural odor contains no more than *K*≪*N* of these types. Then, recent results in mathematics show that when *N* is sufficiently large, a small number of linear sensors (about *K*log*N*) could store complete information about natural odors in a similarity preserving manner, provided that the binding affinities of the sensors are statistically random (Candès et al., 2006; Donoho, 2006; Baraniuk et al., 2010). This fact suggests that rather than having strong responses for a specific set of important molecules, a general purpose receptor repertoire should be selected to have molecular affinities that are as disordered as possible—i.e., lacking symmetry—subject to constraints imposed by biophysics and evolution.

Is there evidence for this view? One challenge is that experimental data is typically only available for a small fraction of the receptors and odors relevant to a species, while the relation between disordered sensing and distance preservation is expected for sufficiently large odor-receptor systems. We will meet this challenge in two ways. In this section, we will show that an efficient decoder of linear, disordered codes does nearly as well on Drosophila ORN sensing data, as it would on odors encoded by an optimal Gaussian, random encoder satisfying the theorems of Candès et al. (2006); Donoho (2006), and Baraniuk et al. (2010). Then in the next section we will develop a method to extrapolate experimental sensing data to construct synthetic sensory systems of a realistic size with the natural sensing statistics. We will directly test the connection between similarity preservation and disordered sensing in this extrapolated sensory system.

To this end, we analyzed firing rates of 24 ORN types in *Drosophila* responding to a panel of 110 monomolecular odorants (Hallem and Carlson, 2006). We used this data to model responses to mixtures of odorants that are complex but sparse like natural odors by constructing a firing rate “response matrix” *R* whose entries specify the responses of each ORN to each monomolecular odorant. To do so, we approximated ORN responses to odor mixtures as linear in the response to each odorant which is a reasonable approximation when the receptors are not in a saturated regime (Tabor et al., 2004; Grossman et al., 2008; Fletcher, 2011; Rokni et al., 2014) (see Reddy et al., 2018; Singh et al., 2019, 2021; Zak et al., 2020 for a more complete treatment of the non-linear responses to mixtures). In this model, we defined a complex mixture as a 110-dimensional composition vector $\vec{x}$ whose entries specify the concentrations (measured relative to those used in Hallem and Carlson, 2006) of monomolecular odorants in the mixture. The ORN firing rates $\vec{y}$ can then be modeled as linear combinations of responses to these odorants: $\vec{y}=R\;\vec{x}$.

To construct each mixture composition vector $\vec{x}$, we set a small number *K* of its elements to be non-zero (where *K* specifies the complexity of the mixture). The values of these non-zero entries were chosen randomly and uniformly between 0 and 2. We then attempted to decode composition vectors ($\hat{x}$) from responses $\vec{y}$ using an efficient algorithm for decoding linearly-combined sparse composition vectors, that uses a *L*_1_−norm penalty to induce sparsity (Candès et al., 2006; Donoho, 2006; Candès and Plan, 2009). We deemed the result a failure if the average squared difference between components of the decoded ($\hat{x}$) vs. original ($\vec{x}$) composition vectors exceeded 0.01, and defined *decoding error* as the failure probability over an ensemble of 500 odor mixtures $\left\{\vec{x}\right\}$. We checked that our findings are robust to different choices of failure threshold used to assess decoding error (Supplement IIIE, Figure 10 in Supplementary Material). Note that even the efficient *L*_1_ decoding algorithm can be computationally intensive (see Rozell et al., 2008 for comments on neural plausibility), and that we have chosen a threshold for successful decoding that is more stringent than typically necessary for animal behavior. The purpose of our analysis is hence not to suggest that animals use this particular method of decoding, but rather to demonstrate that the necessary information for decoding is present in the receptor responses.

**Figure 2:**
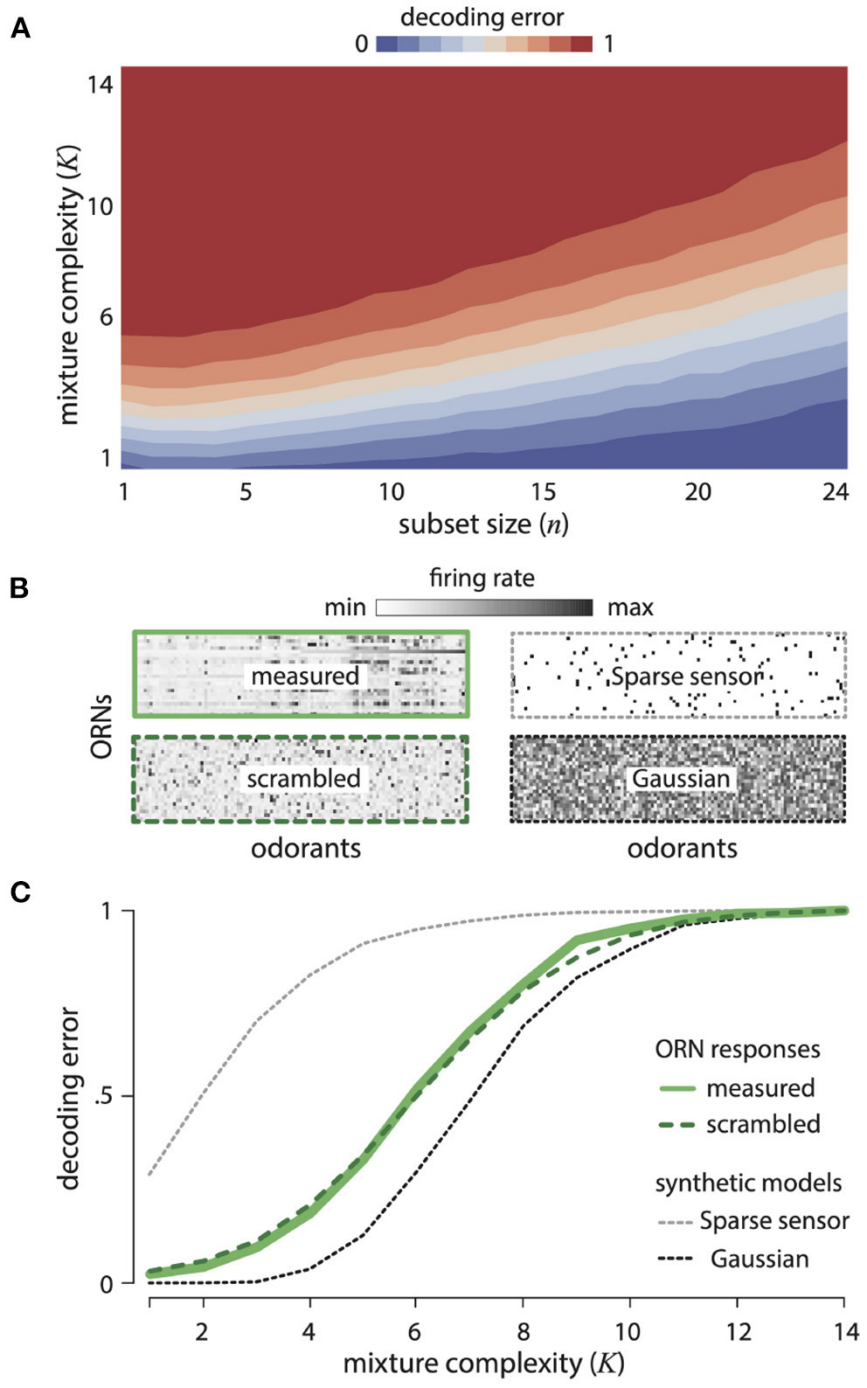
Disordered sensing by ORNs enables accurate decoding of complex mixtures. **(A)** Error in decoding mixture composition from subsets of ORN responses, as a function of mixture complexity *K* (i.e., number of mixture components) and ORN subset size *n*. Results are averaged over 500 odor mixtures of a given complexity, and 50 subsets of a given size. **(B)** Response matrices for *Drosophila* ORNs (measured and scrambled), sparse-sensor and Gaussian models (see text for details). **(C)** Error in decoding complex mixtures from 24 ORNs as a function of mixture complexity *K*, shown for ORN responses (solid green), a scrambled version of ORN responses (dashed green), and two idealized models (the Gaussian random model, dashed black, and the sparse-sensor model, dashed gray). Results are averaged over 500 odor mixtures of a given complexity. Results from scrambled, Gaussian, and sparse-sensor models are additionally averaged over 100 model instantiations.

Figure 2A shows the decoding error for varying mixture complexity *K* and numbers of ORN types. Performance improves with increasing number of ORNs and decreasing mixture complexity. We compared the decoding error obtained from the measured ORN responses to two idealized alternatives: (1) a Gaussian random model, in which each ORN responds randomly to different odorants (with the overall mean and variance matched to data), and (2) a generalized sparse-sensing model, in which each ORN responds (with the same strength) to only five randomly-selected odorants. The Gaussian random model would be an optimal strategy in the limit of many receptors and a large odor space (Candès and Plan, 2009), while the sparse sensing model corresponds to retaining the strongest responses. The *Drosophila* ORNs significantly outperform the sparse-sensing model model and approach the performance of the Gaussian random model (Figure 2C). Quantitatively, 67% of mixtures with 5 or fewer components drawn from 110 odorants can be decoded almost perfectly from the responses of 24 receptors. There are a staggering 100 million such mixtures. Again, this is not to say that the fly brain attempts to reconstruct all of these odors with such an accuracy, but it does say that the receptors contain the necessary information. Our theory also predicts that the olfactory representation of odors does not depend on the details of how specific receptors respond to specific odors, but rather only depends on the broad distribution of responses across many receptors and many odors. We tested this prediction by scrambling the *Drosophila* response matrix (Figure 2B) with respect to both odors and receptors and indeed found identical decoding performance (Figure 2C).

**Figure 3:**
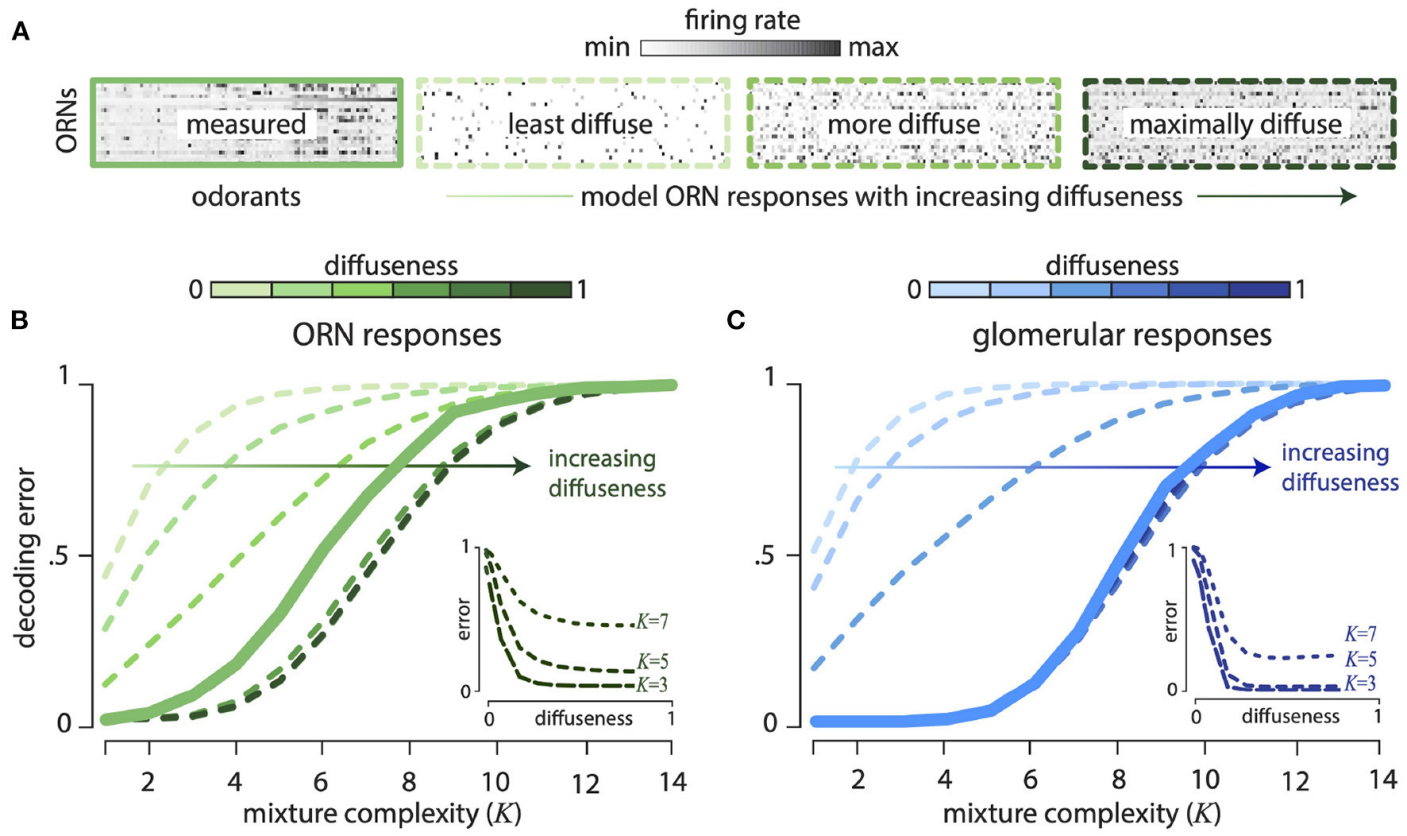
Weakly responding ORNs and glomeruli are informative about odor mixture composition. All results computed for 24 ORN types responding to 110 monomolecular odorants and their mixtures. **(A)** Firing rate response matrix measured from *Drosophila* ORNs (left, solid green), and for increasingly diffuse model response matrices (right, dashed green; “diffuseness” = fraction of largest responses kept). Model responses are constructed by thresholding measured responses and then scrambling the response matrix. **(B)** Error in decoding from ORNs decreases systematically as diffuseness increases—hence weak responses are informative. Results shown as a function of mixture complexity (*K* = number of odor mixture components). **(C)** ORN responses are divisively normalized to produce responses in the glomeruli of the Antennal Lobe. Thresholding and scrambling these responses produces sensing models with different degrees of diffuseness. Error in decoding from glomeruli decreases systematically as diffuseness increases. (Insets): The insets show decoding error as a function of the diffuseness parameter for fixed values of mixture complexity (*K* = 3, 5, 7). For the plots, the models with varying diffuseness are averaged over 100 randomly scrambled model response matrices. Decoding error is measured as the probability of decoding failure over an ensemble of 500 randomly chosen odor mixtures of a given complexity.

Our theory also predicts that the olfactory code spreads information across all receptors, so that even weak responses are informative. To test this comprehensively, we thresholded the *Drosophila* response matrix to keep only a fixed fraction—“diffuseness parameter” *f*—of the strongest responses setting the rest to zero. So a diffuseness value of *f* = 1.0 means we retain all responses, whereas a diffuseness value of *f* = 0.5 means that we retained the strongest 50% of all responses. We, then scrambled the odor identities for each receptor to create receptor responses with the same thresholded distribution. Figure 3A shows the *Drosophila* ORN response matrix, along with model response matrices with increasing diffuseness. Figure 3B shows the decoding error as a function of mixture complexity *K* (number of non-zero components in each mixture) for varying diffuseness. We see that decoding error decreases systematically as diffuseness increases, showing that weak receptor responses are informative about odor mixture identity.

### 2.2. Similarity Preservation in Extended ORN Datasets

**Figure 4:**
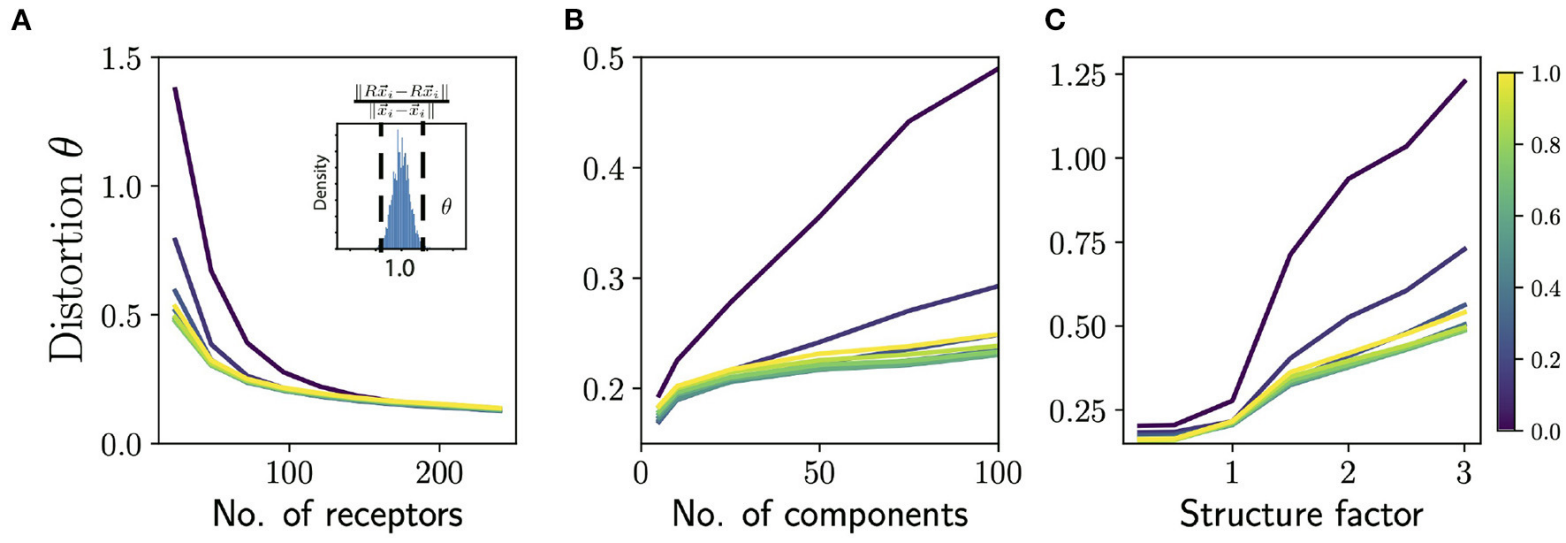
Distortion in similarity of odors by ORN sensing. **(A)** Average distortion measure θ (averaged over odors and responses; see text) as a function of number of receptors for odors with 50 components. Note that this is higher than the number of components in typical natural odors. Different colored lines correspond to different values of “diffuseness” prescribing the fraction of largest ORN responses to be retained in sensing. (*Inset*): Histogram of ratio of pairwise distances between odor composition vectors and their corresponding ORN representations. The distortion measure θ measures how narrowly concentrated this distribution of these ratios is around 1.0. **(B)** Distortion measure θ as a function of number of components in the odors for 48 receptors (~50 is a lower estimate for the number of receptors in *Drosophila*). Note that identifying >50 component odors with ~50 receptors is a hard challenge, and we want to emphasize here the effect of the diffuseness rather than the absolute performance. **(C)** Distortion measure θ as a function of the structure factor for 48 receptors and 50 component odors. The structure factor is a parametric way of controlling the structure in the response matrix by varying the rate of decay of the eigenvalues of the correlation matrix (see Supplement IIIC in Supplementary Material for details). A structure factor of 1.0 corresponds to responses that are as structured as the *Drosophila* dataset and higher values correspond to a faster decay of eigenvalues of the correlation matrix, and thus more structure. For all the panels the total odor space consisted of 1,000 odorants; increasing the size of this space did not seem to have an appreciable effect on the distortion measure.

To judge how well the olfactory code preserves similarity and difference between odors, we can complement the decoding analysis by directly comparing the Euclidean distance between the composition vectors corresponding to two mixtures $\left||{\vec{x}}_{i}-{\vec{x}}_{j}|\right|$ to the distance between the responses they elicit $\left||R{\vec{x}}_{i}-R{\vec{x}}_{j}|\right|$. In order to compare these distances on common grounds, we mean-center the columns of *R* and normalize so that every column has unit norm. We then measure the degree of distortion in similarity by measuring the threshold θ for which 95% of the pairwise distances are distorted by less than θ (see Figure 4A inset). Specifically, θ is defined as


(1) $$\begin{array}{l} P\left(\left|\frac{\left\|R{\vec{x}}_{i}-R{\vec{x}}_{j}\right\|}{\left\|{\vec{x}}_{i}-{\vec{x}}_{j}\right\|}-1\right|>\theta \right)=0.05 \end{array}$$

Note that the mean-centering and normalization of *R* prohibit trivial ways of increasing the distortion, like transformations that globally scale the length of the vectors. We also note that this distortion measure has an asymmetry between transformations that distort relations by *decreasing* the distance and transformations that *increase* the distance, because there is a lower bound of zero on the distortion. The interpretability of this measure nonetheless makes it an useful complement to the decoding analyses above. Using the distortion measure, θ, we can assess how similarity preservation is affected by (i) mixture complexity; (ii) sparsity of responses; and (iii) structure/disorder in responses.

Even though this distortion measure intuitively captures a notion of similarity preservation, it can display high variability on small sensing matrices which have a higher likelihood of being statistically atypical. Even for Gaussian matrices—which are known to be optimal for preserving similarity of sparse vectors—the distortion measure shows substantial variability when the size is similar to that of the *Drosophila* dataset we are using (Supplement IIIB, Figure 7 in Supplementary Material). Therefore, to use this measure to study similarity preservation we need larger datasets. Thus we sought a way of synthetically producing a sensory system that extrapolates the statistics of the experimental data, but with many more odors and receptors.

To study the effect of the interventions on larger sensing matrices, we developed a novel method to generate extended ORN response matrices along both the receptor and odor dimensions. We first observed (Supplement IIIC in Supplementary Material) that the normalized logarithm of the responses of the *n*_*rec*_ = 24 receptors in the *Drosophila* dataset to a given odor is well-approximated by a high-dimensional Gaussian distribution which captures the correlation structure between receptors. We use this observation to model the responses as a Gaussian in the log-response space. Specifically, as described in Supplement IIIC (Supplementary Material), we first create an extended (by a factor *F*) repertoire of *F*·*n*_*rec*_ receptors by generating an extended correlation matrix which replicates the original receptor-receptor correlation matrix *F* times along the diagonal blocks. We then randomly rotate this larger covariance matrix, and use this correlational structure to sample vectors. Finally, an exponential transform generates simulated responses to odors. This procedure allows us to generate response matrices of size *Fn*_*rec*_× *n*_*odors*_.

Using the extended sensing matrix generated in this way, we measured similarity distortion as a function of the number of receptor types and odor complexity (Figures 4A,B) We also varied the sparsity of ORN responses, by controlling the “diffuseness”, a number ranging between 0 and 1 which measures the fraction of largest receptor responses which are retained, with the rest thresholded to zero.

We see that the distortion θ decreases as we add more receptors (Figure 4A) and by the time we reach 150 receptors most of the gain in performance is achieved for odors with odors with ~50 components. Note that most natural odors are not this complex, and the typical repertoire size in animals is 100s of receptor types. Interestingly, the dependence of the distortion measure θ on the diffuseness is sharp, with most the of the gains realized once the diffuseness reaches around ~ 0.2–0.3. Indeed, Prior work has also argued that odor information can be read out sufficiently accurately if each receptor binds to ~ 5–15 % of odorants (Singh et al., 2021). The distortion θ also increases for more complex odor mixtures (Figure 4B) and again we see that very sparse responses (very low diffuseness) lead to worse performance, but a diffuseness of ~ 0.2–0.3 achieves similar levels of distortion as full dense response. This is also consistent with the decoding analysis in the previous section (see Figure 3B, inset), which suggests that a diffuseness of ~ 0.2–0.3 would provide much of the information gains.

Finally, we studied what happens if we parametrically introduce structure in the responses. We do this by parametrically controlling the rate of fall-off of the eigenvalues of the receptor-receptor correlation matrix. A steeper fall-off means there is more redundancy in the receptor responses and thus the response matrix will be more structured (see Supplement IIIC in Supplementary Material for details of the method). According to our general the theory, introducing structure should diminish the ability to preserve similarity. When we systematically increase structure in the responses, we see in Figure 4C that the level of distortion θ grows with structure as expected from the theory.

### 2.3. Reading Out Odor Valence From ORN Responses

**Figure 5:**
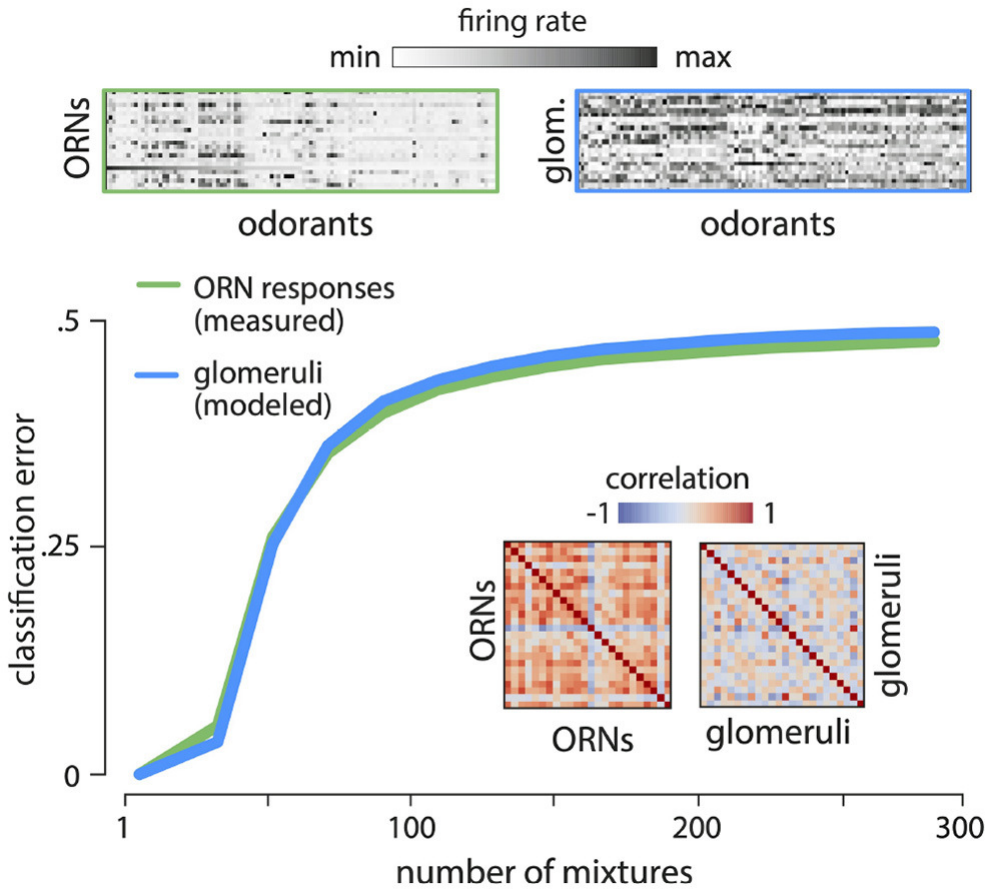
Reading out odor valence from ORN responses. Divisive normalization of ORN responses decorrelates the responses (top - response matrices; inset - correlation matrices). ORN responses and modeled glomerular responses are not readily usable for flexible classification tasks. Error in classifying randomly labeled “appetitive” or “aversive” mixtures from responses of ORNs (green) and glomeruli (blue) quickly approaches chance as the number of mixtures increases. Results are shown for two-class separability of 5- component mixtures, averaged over 100 different ensembles of odor mixtures, and 100 labelings into appetitive and aversive classes.

We saw above that a combinatorial code that employs disordered sensing is an efficient way to compress a vast odor space into a smaller response space. However, *Drosophila* ORN responses are noticeably structured and have a more clustered distribution of firing rates than, e.g., the Gaussian random model (Figure 2B). These correlations, perhaps arising from similarities between odorant binding sites or between receptor proteins, induce some order in receptor responses. These responses are modified when receptors of each type converge to a second stage of processing in distinct glomeruli of the Antennal Lobe (analogously, the Olfactory Bulb in mammals). As has been described before, this second stage of processing decorrelates the responses by a divisive normalization (Olsen and Wilson, 2008; Olsen et al., 2010; Wiechert et al., 2010) (see Supplement IIIE in Supplementary Material and Figure 5 inset). Assuming, a linear response for mixtures at this stage, we find that the *L*_1_−decoding error improves relative to the ORN responses (Figure 3C). This assumption of linear mixing after the non-linear divisive normalization is not equivalent to performing a (complex) decoding of the non-linear mixture responses, and likely gives a more optimistic estimate of the decoding error. Nevertheless, this analysis can be regarded as estimating the decoding performance achievable in a linearized regime around a fixed odor background. Moreover, as with the ORN responses, scrambling the responses over odors and receptors does not change the performance, again suggesting that only the distribution of responses is important for the odor representation (Supplement IIID, Figure 10C in Supplementary Material). Furthermore, in Figure 3C, we see that as with the ORN responses, diffuseness increases the decoding performance; however, most of these gains are realized with a sparsity of ~0.2.

Another key requirement of a sensory olfactory representation is its ability to support flexible associations between odors and valence that are necessary for driving behavior. To study how well the ORN/glomerular representation supports this ability, we randomly labeled mixtures “appetitive” or “aversive”, and trained a linear classifier to identify these labels from ORN and non-linear glomerular responses (Supplement IIIF in Supplementary Material). The performance was poor (Figure 5C), even though mixture compositions can be accurately decoded from these responses (e.g., Figure 2C). This is fundamentally an issue concerning the dimensionality of the representation, and we know that reformatting the representation into a higher-dimensional form can aid in learning associations (e.g., Cover, 1965). We conclude that although these first stages of processing retain nearly complete information about odor mixtures, this information is not readily usable for flexible learning.

### 2.4. Disordered Projections Reorganize Odor Information to Facilitate Flexible Learning

Although early stages of olfactory processing apparently do not support flexible learning, we know empirically that the representation at the third stage in the pathway *can* support such learning (fly: Heisenberg et al., 1985; McGuire et al., 2001; mammal: Choi et al., 2011). How is odor information reorganized to achieve this?

In both insects and mammals, the transformation from the second to third stage of olfactory processing has two notable features: (i) expansive and disordered projections that distribute odor information across a large number of cells (Sosulski et al., 2011; Caron et al., 2013), and (ii) non-linearities that sparsify responses (Turner et al., 2008; Stettler and Axel, 2009). As a result, an odor is represented by a sparse pattern of activity distributed broadly across cells in the third stage. We expect from general theory that this transformation should facilitate flexible associations between odor signals and valence (Cover, 1965; Luo et al., 2010; Barak et al., 2013; Babadi and Sompolinsky, 2014). Here, we propose that an additional source of disorder—lack of structure in the connectivity patterns—allows such associations to be learned from small groups of neurons drawn arbitrarily from within the population.

**Figure 6:**
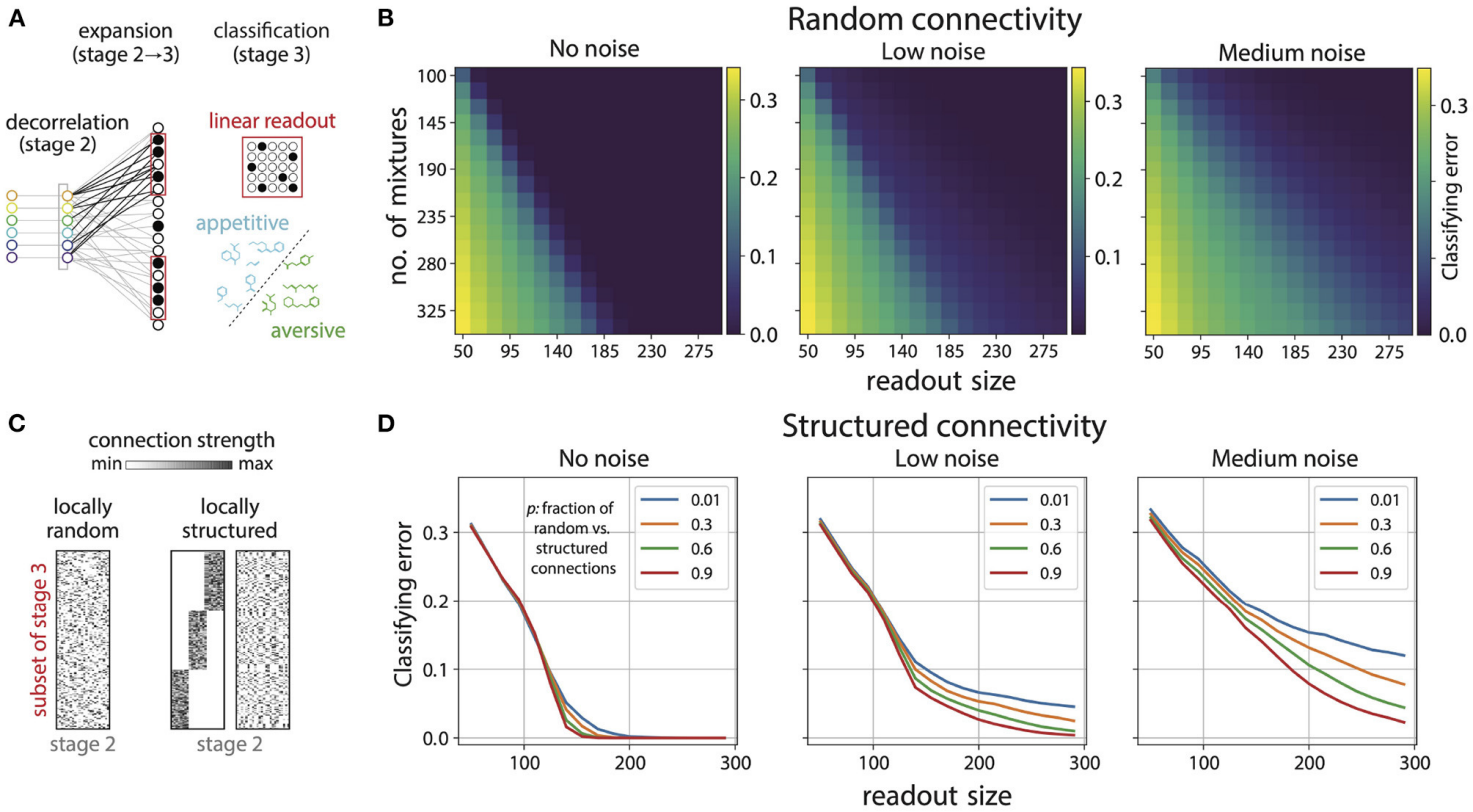
Disordered projections enable flexible learning in the presence of noise. **(A)** Schematic. Small subsets of Kenyon cells exhibit sparse firing patterns in response to odor mixtures. A linear readout neuron can learn to separate arbitrary classes of appetitive and aversive odor mixtures from these responses. **(B)** Small subsets of sparsely active Kenyon cells (here 15% of cells respond) facilitate accurate classification of mixtures. Shown here are plots for the classification error, for random connectivity between the glomerulia and Kenyon cells [see **(C)**], as a function of number of mixtures classified and the number of Kenyon cells used to perform the classification. Noise (introduced in ORN responses; see text) degrades classification performance (left to right). **(C)** We generated random (left) and locally-structured (middle, right) projections from the Antennal Lobe (stage 2) to a subset of cells in the Mushroom Body (stage 3). Local structure was introduced by requiring that a fraction (1/3) of Kenyon cells receive inputs from a fraction (1/3) of all glomeruli (middle). When randomly permuted, the structure is no longer apparent (right). We then parametrically interpolated between the structured and random matrices by varying the probability *p* that a glomerulus could connect to Kenyon cell outside its preferred group. For *p* close to 1.0 the connectivity approaches random (left) and for *p* close to 0 it approaches the matrix on the right. **(D)** Classification error for 230 mixtures as a function of number of Kenyon cells used for readout with locally structured connectivity between glomeruli and Kenyon cells. As expected the classification error decreases with larger readout size; however, in the absence of noise in ORN responses, structure only degrades performance slightly (legend indicates *p*, which controls the amount of structure in connections). As the noise level increases, the degradation in performance for structured connectivity is more pronounced (left to right).

To test this, we simulated the responses of Kenyon cells in the Mushroom Body of the fly to odor mixtures (Figure 6A). We constructed an end-to-end model, starting with linearized mixture responses of ORNs modeled as in Section 2.1, that are divisively normalized in the bulb following (Olsen and Wilson, 2008; Olsen et al., 2010; Wiechert et al., 2010) (see Supplement IIID in Supplementary Material). We modeled each Kenyon cell as receiving inputs from 8 glomeruli selected at random, reflecting empirical estimates (Caron et al., 2013; Litwin-Kumar et al., 2017) (interestingly, fewer or more projections from the bulb yield worse performance; Figure 14 in Supplementary Material). Connection weights were drawn uniformly between 0 and 1 (Figure 6C, left). We modeled long range inhibition by first removing the average response to an ensemble of odors, and then thresholding to eliminate weak responses (Supplement IIIG in Supplementary Material, Luo et al., 2010). This imposed a tunable level of sparsity in the population response. We fixed this sparsity to 15% to match experimental estimates (Turner et al., 2008; Stettler and Axel, 2009). To assess learning, we generated responses to an ensemble of odor mixtures (generated as described above) with 25 components, in the middle of the mixture complexity range reported for natural odors (Yu et al., 2015). Increasing/decreasing the number of mixture components will make the classification the task harder/easier. We then trained a linear classifier to separate responses into two arbitrarily-assigned classes (Supplement IIIF in Supplementary Material). We defined *classification error* to be the fraction of mixtures that are incorrectly labeled by the classifier, averaged over 100 ensembles of mixtures and 100 labeling of each ensemble into appetitive/aversive classes.

We found that small subsets of Kenyon cells can facilitate accurate classification of mixtures (Figure 6B). For instance, 230 mixtures can be classified almost perfectly using any subset of 150 Kenyon cells (Figure 6B, left); there are 23 active Kenyon cells in a typical population of this size. Classifying the same number of mixtures from the ORN/AL responses yields poor performance (Figure 5). Moreover, since the connectivity between Kenyon cells and the glomeruli was taken to be random any typical subset will be equally good for classification [see histogram in Figure 14A (Supplementary Material)]. Increasing the number of cells used as a readout provided no significant benefit beyond a certain point, and decreasing the average sparsity of responses also had minimal effect on the performance (Figure 14 in Supplementary Material). These results suggest that the disordered projections from the second to the third stage of the olfactory pathway reorganize the sensory representation so that associations can be learned from small subsets of sparsely active neurons. Furthermore, these associations can be learned from arbitrary subsets of a given size, suggesting that the information is redundantly represented across in the third stage, i.e., Mushroom Body or olfactory cortex.

Classification performance could deteriorate markedly with noise in the sensory representation. Hence, we probed the effect of variability in the ORN responses on classification performance on the basis of Kenyon cell responses. To do this, we added Gaussian noise with varying standard deviation to the ORN responses (σ_*low*_ = 0.1, σ_*med*_ = 0.3). As expected noise degrades performance, albeit in a graded way (see boundary between good and poor classification in Figure 6B, left to right). We then examined how the variability in responses interacts with the connectivity pattern from the Antennal Lobe to the Mushroom Body. To do this, we introduced local structure in the projections from the Antennal Lobe to the Kenyon cells in the Mushroom Body (Figure 6C, right). Within any chosen subset of Kenyon Cells, we required that a certain fraction received preferential inputs from some glomeruli (in both cases, the fraction was taken to be 1/3). In doing so, we constrained the overall distribution of connection strengths to match those used to generate disordered connectivity (Supplement IIIH in Supplementary Material). This ensured that as a whole, each subset of Kenyon cells sampled all glomeruli, and any differences in performance were guaranteed to arise purely from differences in local connectivity patterns. We then parametrically interpolated between this structured connectivity and random connectivity by varying the probability *p* that a Kenyon cell in the subset could connect to any glomerulus and not just the ones in its preferred group. So, *p* close to 1.0 would give nearly random connectivity and *p* close to zero would give connectivity as shown in Figure 6C, right.

In the absence of ORN response variability, the effect of local structure in the connectivity on the classification performance was small but present, with more structure giving slightly worse performance (Figure 6D, left). As the level of neural variability was increased we saw that more structured connectivity (lower values of *p* as indicated in the legend) gave substantially worse performance (Figure 6D, middle, right). This effect increased with higher variability. Performance degrades with more structure because structured matrices have a sharper fall-off of their singular values, and thus concentrate most of the input power into a small subspace spanned by the corresponding singular vectors. The net consequence of this is higher variability in the most active neurons in the mushroom body for structured matrices compared to random ones (see Supplement IIIH, Figures 12, 13 in Supplementary Material for more details). These results suggest that the disorder in the connectivity between Antennal Lobe and Mushroom body is beneficial for learning flexible associations in the presence of repsonse variability, and any hidden structure hurts classification, with the effect becoming more pronounced with increasing variability.

## 3. Discussion

We propose a new role for *disorder*—or the lack of symmetry—in building sensory representations of sparse, high-dimensional stimuli that are accurate, compact, and flexible. This feature of the olfactory system stands in contrast to other sensory systems like vision, where the neural responses mirror the symmetry/structure observed in the external stimuli to form efficient and compact representations. We argue that this view explains key organizational and functional features of the olfactory system, where disorder plays two key roles: (*i*) diffuse sensing by olfactory receptors serves to compress sparse, high-dimensional odor signals into compact neural representations, and (*ii*) disordered expansion from the Antennal Lobe to the Mushroom Body serves to reformat these representations for flexible learning. This paradigm exploits a key feature of natural odor signals—sparsity—to overcome a bottleneck in the limited number of olfactory receptor types. We used a combination of data and modeling to provide evidence for this paradigm in fruit fly. Olfactory circuits in mammals show very similar anatomical and functional motifs, including broad receptor tuning (Saito et al., 2009) and apparently disordered projections to the cortex (Sosulski et al., 2011). This convergence between distant species suggests that disorder could serve a computational function in the architecture of early olfactory circuits.

### 3.1. The Logic of Olfactory Receptors

Our theory predicts that general-purpose olfactory receptors should be selected for diffuse binding to many odorants, and not for the strong and specific binding often seen in biochemical signaling. An alternative view suggests that receptors should be adapted to bind selectively to molecules in particular odor environments or ecological niches (Carey et al., 2010; Zwicker et al., 2016). These alternatives can be separated in experiments that measure the affinities of olfactory receptors to very large panels of odorants with varying ethological relevance. We predict that the typical receptor will have a diverse range of binding affinities across a broad array of odorants, with a statistically similar spread across molecules that both do and do *not* have immediate ethological importance. Likewise, we predict that receptors in different species, even related ones, will typically have broadly different distributions of binding affinities, with similarities arising from biophysical constraints of olfactory receptors and not from properties of ecological niches. In addition, as a whole, the receptor repertoires of different species will show similar coverage across the space of odorants. This strategy resembles that of well-adapted immune repertoires, where different antibody distributions achieve similar coverage of the same pathogen landscape, as predicted theoretically (Mayer et al., 2015) and observed in experiment (Venturi et al., 2008; Elhanati et al., 2014; Thomas et al., 2014).

### 3.2. The Computational Role of Expansive and Disordered Projections

While this work provides evidence for the role of disordered sensing in the *compression* of odor information, it also adds to a growing body of work on the computational role of *expansion via* disordered neural projections. Expansive projections are known to make classification easier (Cover, 1965; Luo et al., 2010; Barak et al., 2013), and the computational benefits of this expansion can be further improved by Hebbian learning (Babadi and Sompolinsky, 2014) and by sparse connectivity (Litwin-Kumar et al., 2017). We have argued here that the primary purpose of the expansion from the second to the third stage of olfactory processing is to reorganize a highly compressed representation of odors produced by disordered sensing by the receptors. By contrast, other studies have proposed that this expansion could itself implement a form of odor signal compression (Krishnamurthy et al., 2014; Stevens, 2015), or even a direct encoding of odor space (Zhang and Sharpee, 2016; Kepple et al., 2019) (in one case requiring strict relations between the expansion and ORN responses; Zhang and Sharpee, 2016). We found no evidence that expansive projections implement a form of compression, nor do we find evidence to support the direct representation of odor composition in Kenyon cell responses. Rather, we found evidence that the expanded representation is organized to support flexible learning of categories (Choi et al., 2011; Gruntman and Turner, 2013) from modest subsets of Kenyon cells. Anatomical evidence in fly indeed suggests that each olfactory readout neuron samples only a fraction of the Mushroom Body (Schroll et al., 2006) while still allowing formation of complex associations (Fiala, 2007). Our view is also consistent with abstract theory showing that sparsely firing binary neurons with “mixed selectivity” permit both discrimination between, and effective generalization from, complex overlapping binary inputs (Barak et al., 2013; Rigotti et al., 2013). Our work can be viewed as additionally showing that *receptor* neurons with “mixed selectivity” effectively compress high dimensional sensory information, while subsequent “mixed *sampling*” of these responses reformats them for flexible learning by a simple readout. It would also be interesting to understand the relationship between our results and the finding in Singh et al. (2021) that receptors that *do not* respond to an odorant are particular informative about its identity.

### 3.3. Implications for Behavior

Conceptually, our key idea is that disorder in the olfactory system is a fundamental adaptation to the intrinsic complex structure of the world of smells. We predict, distinctively, that odor information is distributed in both weak and strong responses across the entire ensemble of olfactory receptor types, and that this is important for complex discrimination tasks. An alternative view suggests a “primacy” code where only the earliest or strongest responses are relevant for behavior (Wilson et al., 2017; Dewan et al., 2018; Kepple et al., 2019). We have shown (Figure 3) that an encoding scheme that retains only the strongest responses contains much less information about complex mixtures than does a scheme that retains both strong and weak responses. Because of this, we expect that our view can be separated from the primacy code in behavioral experiments that vary the complexity of discrimination tasks, e.g., by increasing the number of odors, the number of mixture components, and the degree of overlap between mixture components. Given knowledge of responses to individual odorants, our theory quantitatively predicts the decline of behavioral performance with task complexity (e.g., Figures 2–5). Likewise, our theory predicts how the relationship between behavioral performance and task complexity will vary as a function of information content in the olfactory pathway. This information content can be experimentally manipulated by creating genetically-impoverished or enhanced receptor repertoires, or optogenetically activating Kenyon cells to simulate structured projection patterns from the Antennal Lobe.

### 3.4. The Role of Valence and Context

Our analysis has focused on ways in which the olfactory system can faithfully represent and then recover the similarities and differences between odors seen as concentration vectors in a high-dimensional space of odorants. In fact, in vertebrates behavioral valence and context are known to change both perceptual similarity of odors, and representational similarity in cortex. These changes may be entrained by the extensive feedback that is present from the central brain to the olfactory bulb. Interestingly, recent work (Tavoni et al., 2021; Kersen et al., 2022) suggests that these feedback effects of context and valence need not be structured, and instead can also be disordered. That is, each behavioral context can effectively be represented as a random vector of feedback to the circuit elements of the bulb. It would be interesting to understand how such disordered feedback interacts with disorder in the ORN encoding map and feedforward projections to cortex that we have discussed in this paper, while also including non-linear models of ORN response such as those in Singh et al. (2019).

### 3.5. Looking Ahead

Testing these predictions requires a movement away from simple paradigms involving small mixtures and pairwise discrimination, toward far more complex tasks that are reflective of life in the real world. Methodologically, this shift has begun occurring in the study of vision. We have argued here that in olfaction, this shift is even more critical—the functional logic of the sense of smell can only be understand by taking into account the complexity of the real odor world.

## Data Availability Statement

The data analyzed in this study is subject to the following licenses/restrictions: the data is available by request from the original authors. Requests to access these datasets should be directed to Elissa Hallem, ehallem@ucla.edu.

## Author Contributions

KK, AH, AW, TM, and VB conceived of the project, carried out the research, and wrote the paper. All authors contributed to the article and approved the submitted version.

## Funding

This research was supported by Simons Foundation Mathematical Modeling in Living Systems Grant 400425 to VB. KK is supported by a C. V. Starr fellowship and a CPBF fellowship (through NSF PHY-1734030).

## Conflict of Interest

The authors declare that the research was conducted in the absence of any commercial or financial relationships that could be construed as a potential conflict of interest.

## Publisher's Note

All claims expressed in this article are solely those of the authors and do not necessarily represent those of their affiliated organizations, or those of the publisher, the editors and the reviewers. Any product that may be evaluated in this article, or claim that may be made by its manufacturer, is not guaranteed or endorsed by the publisher.
