# Supplementary material for: Disorder and the Neural Representation of Complex Odors
Source: Front Comput Neurosci. 2022 Aug 8;16:917786. doi: 10.3389/fncom.2022.917786 (PMC9393645; doi:10.3389/fncom.2022.917786)
Supplement: Supplementary file 1 [file Data_Sheet_1.pdf]

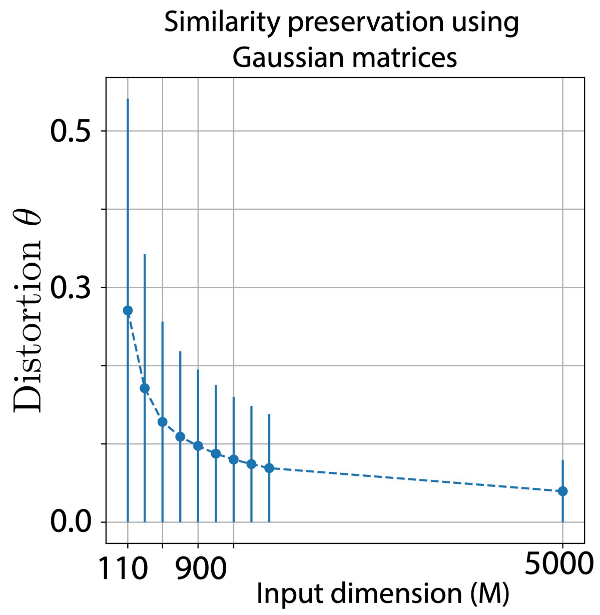

FIG. 7. **Pairwise distance distortion using Gaussian sensing matrices** The pairwise distance distortion threshold, θ as a function of the input dimension for 25-sparse vectors projected using a $N \times M$ Gaussian matrix with i.i.d entries with zero mean and variance $1/N$. The ration N/M is fixed to be 24/110.

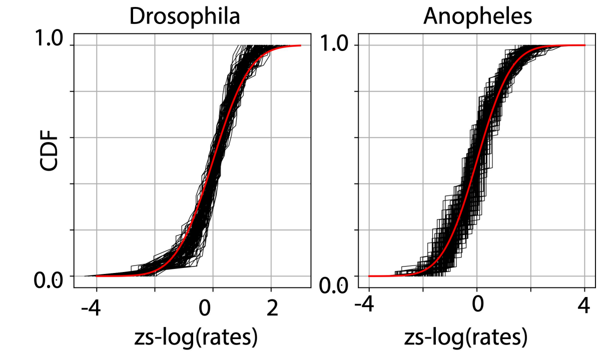

FIG. 8. **log-normal distribution of rates** Each black line represents the cumulative distribution of the log-rates for the repertoire for an odorant after subtracting the mean and normalising by the variance (no. of lines is equal to the number of odorants tested). The red line is the cumulative distribution for a standard normal distribution. Data are for *Anopheles* [46] (right) and *Drosophila* [45] (left)

III. SUPPLEMENT

A. Decoding odor composition

To reconstruct $\vec{x}$ from measurements $\vec{y} = R\vec{x}$, we used the Iteratively Reweighted Least Squares (IRLS) algorithm [80] to find the vector that minimizes the L_1 norm of $\vec{x}$ subject to the constraint $\vec{y} = R\vec{x}$, with 500 maximum iterations and a convergence tolerance (in norm) of 10^{-6}.

B. Distortion using Gaussian sensing matrices

We first generate 200 vectors $\vec{x}_i$ with 25 non-zero elements uniformly sampled between 0 and 2. We then project these vectors by a matrix R of dimension $N \times M$ where the ratio between N and M is fixed such that $N/M = 24/110$. The elements of matrix are independent samples from a Gaussian distribution with zero mean and variance $1/N$. The distortion measure θ is defined as in eq. 1.

C. Matrix extension and controlling structure

The starting point for generating extended ORN response matrices is a log-normal model for the rates. Fig. 8 shows that the logarithm of the responses of all the receptors to all odors agrees well with a Gaussian distribution once we subtract the mean and normalize by the standard deviations of the responses for that odor (z-scoring). Thus in the space of log-rates, we model the responses as a multi-dimensional Gaussian described with the same mean and covariance between receptors as in the *Drosophila* data. To extend the response matrix along the odorant dimension we simply sample from this multi-dimensional Gaussian and exponentiate the Gaussian sample to get the rates. In order to extend along the receptor dimension, we want to create new receptors which share some features of the original data but are not simply duplicates. To do this, if we want to expand the number of receptors by a factor F, say, we create a new covariance matrix (of size $Fn_{rec} \times Fn_{rec}$) which is a randomly rotated version of a block diagonal matrix which has the receptor covariance matrix of the Hallem & Carlson data replicated F times along the diagonal. We generate a synthetic rate matrix by generating Gaussian samples using this covariance matrix and then exponentiating the samples to get the rates. There are small number of outlier rates which we finally set to the maximum value in the *Drosophila* dataset.

To parametrically control the amount of structure in the dataset, we use the fall-off of the eigenvalues of the covariance matrix of the log-rates as a measure of structure – faster fall-off indicates most of the variance is along a few directions and thus corresponds to more structure since there is more redundancy in the receptor responses. We vary this fall-off while keeping the overall variance the same. Specifically, we fit an exponential $\alpha + e^{-\beta * r}$ to the eigenvalue fall-off in the *Drosophila* dataset, where r is a number between 0 and 1 which specifies the normalised rank of the sorted eigenvalue. The choice of this fit was motivated by observing an approximately exponential form for the intial fall-off of eigenvalues. To increase/decrease the structure we increase/decrease β re-

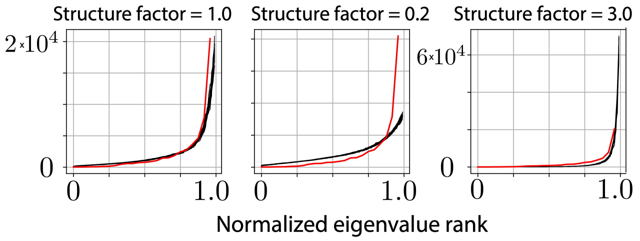

FIG. 9. **Covariance matrix eigenvalues for structured matrices** Eigenvalues of the covariance matrix for the extended response matrices (black lines) compared with the eigenvalue fall-off for the *Drosophila* dataset (red line). A structure factor of 1.0 implies a fall-off similar to the *Drosophila* dataset

spectively by multiplying it with a *structure factor*, while keeping the overall sum of the eigenvalues the same (for a given no. of receptors and odors). Fig. 9 shows the eigenvalue fall-off for three different structure factors – a structure factor of 1.0 indicates a fall-off similar to the *Drosophila* dataset, and higher values indicate more structured responses.

D. Robust decoding from ORN responses

In the main text, we considered a simple linear model of the responses of 24 ORN types in *Drosophila* responding to odor mixtures. Specifically, we extracted a firing rate matrix R from the data in [45] (i.e. R_{ij} is the response of receptor i to odorant j), and we assumed that the response to a mixture could be written as a linear combination of responses to single odorants. We defined a mixture by the composition vector x whose elements specify the concentration of individual odorants in the mixture. The ORN firing rates y could then be written as $\vec{y} = R\,\vec{x}$. We then attempted to decode composition vectors $\vec{x}$ from responses $\vec{y}$ using the optimal algorithm of [55, 80]. We regarded the reconstruction as a failure if the average squared difference between components of the reconstructed odor vector and the original exceeded 0.01. Decoding error was defined as the failure probability over an odorant mixture ensemble. This criterion for successful reconstruction is equivalent to saying that the reconstruction $\hat{x}$ of the odor composition vector $\vec{x}$ fails if the norm of the difference $\|\vec{x} - \hat{x}\|$ exceeds a tolerance parameter of $t = 1.1$ (here we used the fact that the odor composition vector $\vec{x}$ has 110 components). To test the robustness of our conclusions we varied this tolerance parameter ten-fold, and found that the decoding error curves were largely unchanged (Fig. 10A). Qualitatively, we observed this robustness because the decoding of odors tends to either succeed very well, or fail very badly. As a result, a broad range of criteria for defining a successful reconstruction will give similar measures of decoding error.

According to our general theory, and the results of [55, 81], the quality of the olfactory code should not depend on the details of how specific receptors respond to different odorants. Rather, the key determinant should be the overall distribution of responses. To test whether this is the case, we scrambled the receptor and odorant labels in the ORN response matrix (top inset in Fig. 10B), thus constructing an artificial response matrix with the same overall *distribution* of firing rates, but with no odor- or receptor-dependent correlations (second inset in Fig. 10B). We found that decoding performance was essentially identical when using the scrambled and unscrambled response matrices (Fig. 10B), consistent with the notion that the olfactory system seeks to employ disordered and unstructured sensing. Interestingly, separate scrambling of the receptor labels and odor labels either improved or degraded the decoding, presumably because such scramblings removed correlations that were either detrimental or beneficial for decoding (Fig. 10B). These opposite effects compensated each other when the sensing matrix was fully scrambled.

E. Divisive normalization in the Antennal Lobe and decoding glomerular responses

In the Antennal lobe, a network of inhibitory interneurons reorganizes the receptor responses for transmission downstream [59]. In the fly, the inhibitory network is well-described as effecting a divisive normalization [57, 58] that scales the responses of each ORN type in relation to the overall activity of all types . This lateral inhibition in the can be modelled with the following transformation [57]:

$$R_i^{(2)} = \frac{R_{max} \cdot (R_i^{(1)})^{1.5}}{\left[\sigma^{1.5} + (R_i^{(1)})^{1.5} + (m \cdot \sum_i R_i^{(1)})^{1.5}\right]} \qquad (2)$$

where $R_i^{(1)}$ is the response of the ith ORN type, $R_i^{(2)}$ is the response of the ith glomerulus, σ parametrizes spontaneous activity, and m controls the amount of normalization. We use $R_{max} = 165.0$, $\sigma = 10.5$, and $m = 0.05$ [57]. We constructed an artificial glomerular response matrix $R^{(2)}$ by applying this transformation separately to the ORNs responding to each of the 110 odorants studied in [45]. Thus $R_{ij}^{(2)}$ represented the response of the ith glomerulus to the jth odorant.

Applying this transformation to the *Drosophila* response matrix, the glomerular responses become more widely distributed and less correlated than their ORN inputs as has been described before. Does this increased disorder improve the representation of odor information? Because the divisive normalization is nonlinear, we cannot, strictly speaking, use the aforementioned decoding algorithm to evaluate the information content of the glomerular representation. However, we can instead create an artificial benchmark in which mixtures $\vec{x}$ lead to

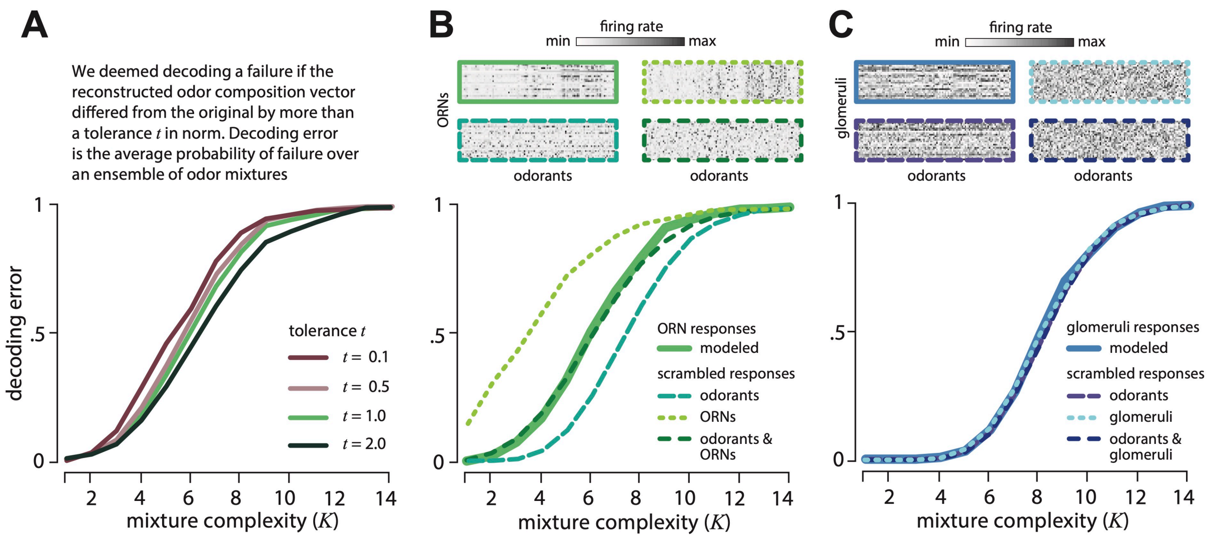

FIG. 10. **Odor decoding from *Drosophila* ORN responses is robust.** (A) Decoding error is robust to ten-fold variations in odor reconstruction tolerance. Mixture complexity = number of component odorants drawn from 110 possibilities. (B) Decoding performance is unchanged after complete scrambling of the *Drosophila* response matrix, because of opposite effects of scrambling receptors vs. odors. Insets: Response matrices showing firing rates for 24 receptors (rows) responding to 110 monomolecular odorants (columns) without scrambling (solid green) and for models randomly scrambling receptors, odorants, or both (dashed green). (C) Decoding performance is unchanged after complete scrambling of the divisively normalized responses in the Antennal Lobe. Separately scrambling receptors or odors also has no effect on performance. Insets: Response matrices showing activity for 24 glomeruli (rows) responding to 110 monomolecular odorants (columns) without scrambling (solid blue) and after scrambling receptors, odorants, or both (dashed blue). Results shown are averages over 100 iterations over model scrambled response matrices. Decoding error is measured as the probability of decoding failure (see text) over an ensemble of 500 randomly chosen odor mixtures of a given complexity.

responses $\vec{y}$ via $\vec{y} = R^{(2)}\vec{x}$, where $R^{(2)}$ represents a matrix of artificial glomerular responses obtained by transforming experimentally measured ORN responses to an odor panel in [45] via divisive normalization. Quantitatively, 67% of mixtures with 7 or fewer components drawn from 110 odorants can be accurately decoded from the responses of 24 glomeruli, while similar accuracy was achieved for mixtures with only 5 components when decoding from ORNs (Fig. 10C). Because the number of possible mixtures increases combinatorially with the number of mixture components, this is a substantial improvement. When we scrambled the identity of odors and receptors, all scramblings left the decoding performance unchanged (Fig. 10C). We thus conclude that after correlations are removed by divisive normalization, the overall distribution of responses is the sole determinant of the quality of the olfactory information representation.

We tested how our results for decoding error would be affected by changing the parameter m, which controls the amount of inhibition in the Antennal Lobe, or the exponent a, which controls the shape of the nonlinearity. In order to simplify our presentation, we study dependence on the parameters of the normalization for two values of mixture complexity: i) $K = 3$, a value where odor reconstruction from Antennal Lobe responses with experimentally-measured parameters is near perfect (see main text), and ii) $K = 7$, a value where a similar reconstruction starts to degrade. We found that in both cases, the experimentally measured values of m and a led to the lowest decoding error (Fig. 11).

F. Linear classification

To measure how well a particular odor representation (responses of ORNs, glomeruli, or Kenyon cells) facilitates learning flexible associations between odors and valences, we randomly split the representation of input mixtures into two classes and then trained a linear classifier (SVM with linear kernel [82]) to classify the inputs.

G. Generating Mushroom Body responses

We took each Kenyon cell to have non-zero connection weights drawn uniformly between 0 and 1 with 8 randomly selected glomeruli (see Results). Then, following [20], we took the input to the ith Kenyon cell, evoked by an odor with glomerular responses $\vec{y}$ in the Antennal Lobe, to be $h_i = \langle \vec{w}_i, (\vec{y} - \langle \vec{\mu}, \vec{y} \rangle \vec{\mu}) \rangle$, where $\langle \cdot, \cdot \rangle$ is an inner-product, $\vec{w}_i$ is the vector of connection strengths, and $\vec{\mu}$ is the average Antennal Lobe response vector over all odors, normalized to unit length. We chose a response threshold so that a fraction f of neurons with inputs h_i

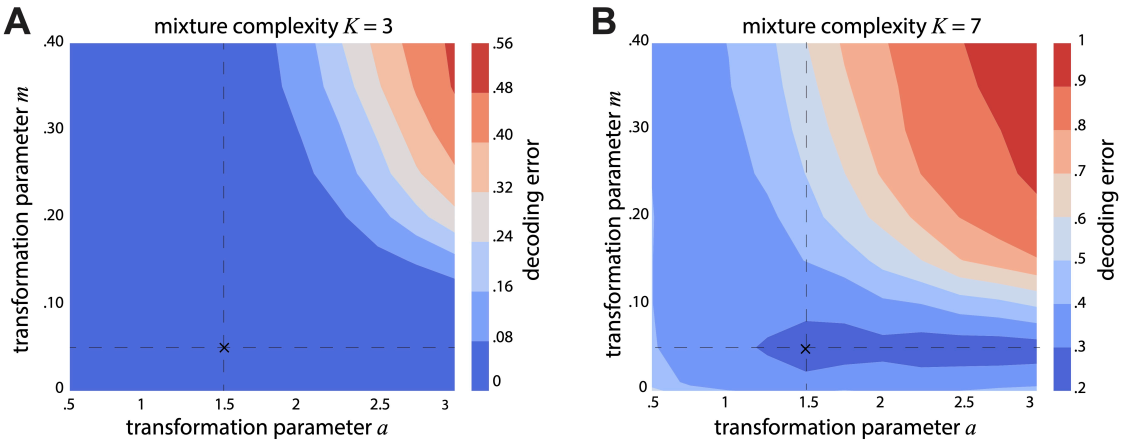

FIG. 11. **The empirically determined divisive normalization in the Antennal Lobe is optimal assuming a linear model for mixtures** Decoding error (see main text for definition) shown as a function of the exponent a, and the inhibition parameter m in the divisive normalization carried out by the Antennal Lobe. Left and right plots correspond to mixtures with $K = 3$ and $K = 7$ components drawn randomly from 110 odorants, respectively. The experimentally measured operating point is indicated by a cross in each plot ($m = 0.05$ and $a = 1.5$). Decoding error (definition in main text) is averaged over 500 iterations of mixture ensembles of a given complexity.

exceeding threshold are considered active, and normalized the thresholded responses so that the maximum firing rate is 5 Hz, on the order of the maximum observed Kenyon cell responses. We averaged results over 100 random choices of connection strengths. The global inhibition required in this model for generating the disordered responses observed in the Mushroom Body [20] could be implemented by the APL neuron which makes inhibitory connections to all the Kenyon cells

H. Structured vs. random connectivity and its interaction with response variability

We constructed structured connectivity matrices between glomeruli in the Antennal Lobe and Kenyon cells in the Mushroom Body by reordering the columns of the corresponding random connectivity matrix so that the two matrices model synapses with the same connection strengths feeding into each Kenyon cell, but they sample different glomeruli. The reordering of the columns was done so that the structured connectivity matrix exhibited a block-diagonal structure as shown in Fig. 6C. For analyses we chose the number of blocks to be 3. We then permuted the rows and columns of the structured connectivity matrix. To parametrically vary the amount of structure between the block diagonal connectivity matrix and the random matrix, we specified the probability p that a Kenyon cell could connect to any glomerulus and not just the ones in its preferred group.

Now, we discuss how the effect of the structured and random projections on the ability to learn arbitrary associations in using Mushroom Body neurons (Fig. 6 B,D). The main reason for the reduced classification performance with the structured projection matrix from the Antennal Lobe to Mushroom Body, relative to the random projection matrix, is due to a higher "effective noise" in the most active neurons. This can be understood by viewing the responses in the Mushroom Body as a linear projection + thresholding of the responses in Antennal Lobe. The effect of increased structure in the projection matrix is to focus the projections from the Antennal Lobe into a smaller subspace. Another way to see this is by considering the fall-off of the singular values of the random and the structured projection matrices. The structured matrix has a steeper fall-off and larger outlier singular values compared to the random projection matrix (Fig. 12, left). A consequence of focusing the Antennal Lobe responses into a smaller subspace is that the elements of the modes corresponding to this subspace will be larger (compare Fig. 12 right, top and bottom panels), and thus response variability in the Antennal Lobe will lead to a larger variability in the tail responses (most active neurons) in the Mushroom Body for the structured projection matrix. This can be seen in Fig. 13 *left*, where the histogram of residual responses (noiseless response minus response with noise) of Mushroom Body neurons is larger in magnitude, on average for the structured projection matrix compared to the random projection matrix.

How does this result depend on the form of sensing employed by the ORNs? Basically, the higher effective-noise property for structured matrices from Antennal Lobe to Mushroom Body will hold regardless of the form of ORN sensing. As an example, in Fig. 13 *right*, we show the residual responses for random Gaussian sensing by the ORNs, and even in this case, the structured matrix leads to larger magnitude residual responses. What changes with the sensing method is the quality of representation of the external input in the ORN and Antennal Lobe

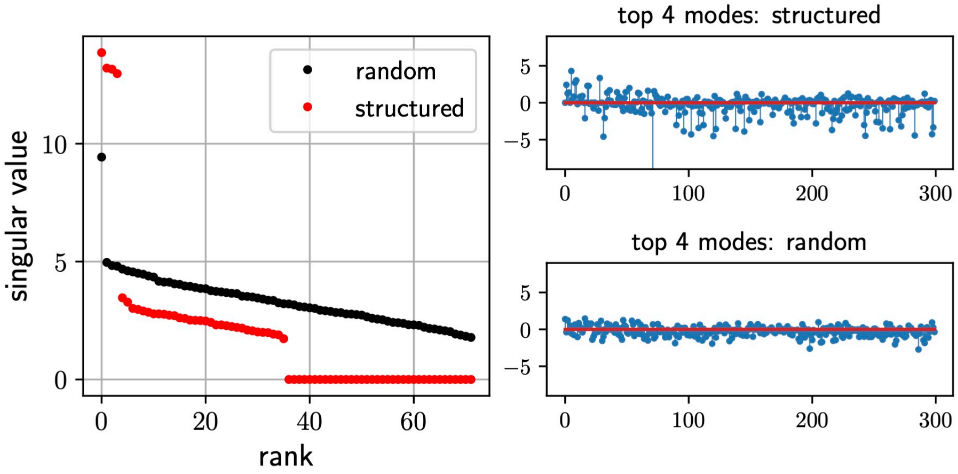

FIG. 12. *Left*: Example plot showing ranked singular values of the random and structured projection matrices from Stage 2 (antennal lobe/bulb) to Stage 3 (cortex/mushroom body). For this example, the size of Stage 2 was 72 and the size of Stage 3 was 300 and there were 4 preferred groups in the structured projection matrix (see main text for the construction of the structured matrix). *Right*: the components of the sum of the first four dominant singular vectors : $\sum_{i=1}^{4} \sigma_i \mathbf{u}_i$, where the full projection matrix P is given by $P = \sum_{i=1}^{70} \sigma_i \mathbf{u}_i \mathbf{v}_i^{\dagger}$. The components of the projection give a sense of how the input power is distributed in the dominant subspace. From the top panel, we see that the structured projection matrix directs more of the input power to the subspace.

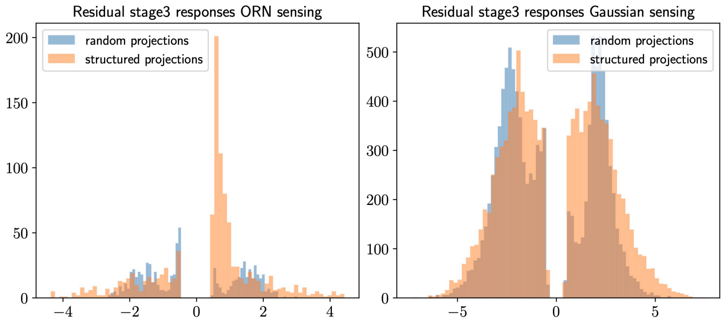

FIG. 13. Histogram of residual responses at Stage 3 that are larger than a threshold (0.5 in this case), for random and structured projection matrices. The residual response is defined as the response at Stage 3 without any noise in Stage 1 responses minus the response at Stage 3 with noise in Stage 1 responses. Larger magnitudes for the residual response means that a classifier trained on noiseless responses will have a higher error for the noisy responses. We see that on average, the structured projection matrix leads to larger magnitude residual responses. The left plot corresponds to ORN-like sensing in Stage 1 (see main text; matrix extension), and the right plot corresponds to a hypothetical random Gaussian sensing at Stage 1. In both cases, the structured projection matrix from Stage 2 to Stage 3 leads to larger magnitude residual responses.

responses. So a poorer sensing method will lead to an overall reduction in the ability to learn arbitrary associations.

I. Classification using Kenyon cells: role of sparsity of responses and connections

Here, we studied the error in a 2-way classification task for 5-component mixtures with varying readout population sizes (n) and the number of Kenyon cells used as readout in the Mushroom Body (details of classification procedure and task in the main text). For a given population size n, increasing the fraction of active neurons f barely changes the classification performance (bottom panel of Fig. 14A). The classification error with a given active fraction f decreases with the number n of neurons being read out (left panel of Fig. 14A). However, there is a law of diminishing returns – excellent performance is achieved for relatively small n, and further increasing the population size makes little difference. The disordered projections from the Antennal Lobe to the Mushroom Body suggest that any subset of a given size should be statistically equivalent. We tested this by comparing the classification error obtained from different subsets of Kenyon cells. The narrowness of the his-

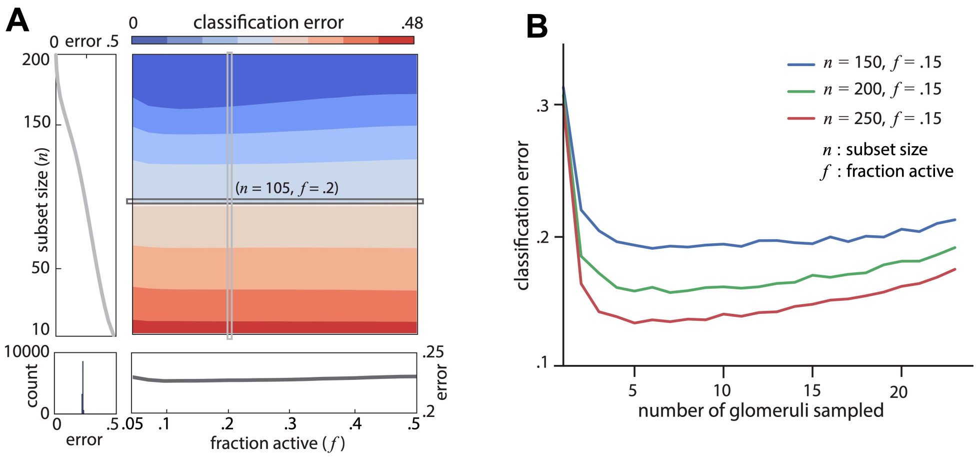

FIG. 14. A) Classification error from responses of model Kenyon cells in the Mushroom Body (MB) for arbitrarily separating 300 5-component mixtures into two classes as a function of the readout size (n) and the fraction (f) of active neurons. The horizontal and vertical sections correspond to $n = 105$ and $f = 0.2$, respectively (section shown in panels below and to the left, respectively). Bottom left panel: histogram of classification errors for 10000 different subsets of size $n = 105$ and $f = 0.2$. The narrowness of the histogram shows that any two subsets of a given size are roughly equivalent for odor classification purposes. B) Classification error at the Mushroom Body as a function of the number of glomeruli sampled by each Kenyon cell. Minimum error is found for sparse sampling of glomeruli. All results shown are averages over 100 iterations over mixture ensembles, 100 labelings into appetitive/aversive classes, and 100 iterations over model connectivity matrices between the Antennal Lobe and Mushroom Body (each using a different instantiation of noise). (See text for details regarding the generation of connectivity matrices and noise.)

togram of classification error for 10000 different populations ($n = 105, f = 0.2$) (lower left panel, Fig. 14A) shows that any subset of a given size is indeed equally good at supporting flexible classification.

We also studied how the classification error (in the presence of ORN response variability) depended on the number of glomeruli sampled by each Kenyon cell in the Mushroom Body. Figure 14B shows the classification error as a function of the number of glomeruli sampled, for three different readout sizes. We see that the classification error initially decreases and then gradually rises as we increase the number of glomeruli sampled. This indicates that there is an optimum for the number of sampled glomeruli. Recent work [21] has examined this question theoretically; here we show results with *Drosophila* data which are consistent with [21].

[1] M. Dunkel, U. Schmidt, S. Struck, L. Berger, B. Gruening, J. Hossbach, I. S. Jaeger, U. Effmert, B. Piechulla, R. Eriksson, *et al.*, "Superscent—a database of flavors and scents," *Nucleic Acids Research*, vol. 37, no. suppl_1, pp. D291–D294, 2009.

[2] E. J. Mayhew, C. J. Arayata, R. C. Gerkin, B. K. Lee, J. M. Magill, L. L. Snyder, K. A. Little, C. W. Yu, and J. D. Mainland, "Drawing the borders of olfactory space," *bioRxiv*, 2020.

[3] K. Touhara and L. B. Vosshall, "Sensing odorants and pheromones with chemosensory receptors," *Annual review of physiology*, vol. 71, pp. 307–332, 2009.

[4] L. B. Vosshall, A. M. Wong, and R. Axel, "An olfactory sensory map in the fly brain," *Cell*, vol. 102, no. 2, pp. 147–159, 2000.

[5] S. Zozulya, F. Echeverri, and T. Nguyen, "The human olfactory receptor repertoire," *Genome biology*, vol. 2, no. 6, pp. 1–12, 2001.

[6] X. Zhang and S. Firestein, "The entire mouse olfactory subgenome," *Nat. Neurosci*, vol. 5, pp. 124–134, 2002.

[7] C. Bushdid, M. O. Magnasco, L. B. Vosshall, and A. Keller, "Humans can discriminate more than 1 trillion olfactory stimuli," *Science*, vol. 343, no. 6177, pp. 1370–1372, 2014.

[8] R. C. Gerkin and J. B. Castro, "The number of olfactory stimuli that humans can discriminate is still unknown," *Elife*, vol. 4, p. e08127, 2015.

[9] B. Malnic, J. Hirono, T. Sato, and L. B. Buck, "Combinatorial receptor codes for odors," *Cell*, vol. 96, no. 5, pp. 713–723, 1999.

[10] M. Stopfer, V. Jayaraman, and G. Laurent, "Intensity versus identity coding in an olfactory system," *Neuron*, vol. 39, no. 6, pp. 991–1004, 2003.

[11] C. F. Stevens, "What the fly's nose tells the fly's brain," *Proceedings of the National Academy of Sciences*, vol. 112, no. 30, pp. 9460–9465, 2015.

[12] Y. Zhang and T. O. Sharpee, "A robust feedforward model of the olfactory system," *PLoS computational biology*, vol. 12, no. 4, p. e1004850, 2016.

[13] H. Saito, Q. Chi, H. Zhuang, H. Matsunami, and J. D. Mainland, "Odor coding by a mammalian receptor repertoire," *Science signaling*, vol. 2, no. 60, pp. ra9–ra9, 2009.

[14] R. C. Araneda, A. D. Kini, and S. Firestein, "The molecular receptive range of an odorant receptor," *Nature neuroscience*, vol. 3, no. 12, pp. 1248–1255, 2000.

[15] M. Bazhenov and M. Stopfer, "Olfactory coding," *Encyclopedia of Neuro-scienceScience Oxford: Academic Press*, vol. 7, pp. 87–94, 2009.

[16] G. Laurent, M. Stopfer, R. W. Friedrich, M. I. Rabinovich, A. Volkovskii, and H. D. Abarbanel, "Odor encoding as an active, dynamical process: experiments, computation, and theory," *Annual review of neuroscience*, vol. 24, no. 1, pp. 263–297, 2001.

[17] L. M. Kay and M. Stopfer, "Information processing in the olfactory systems of insects and vertebrates," in *Seminars in cell & developmental biology*, vol. 17, pp. 433–442, Elsevier, 2006.

[18] G. B. Choi, D. D. Stettler, B. R. Kallman, S. T. Bhaskar, A. Fleischmann, and R. Axel, "Driving opposing behaviors with ensembles of piriform neurons," *Cell*, vol. 146, no. 6, pp. 1004–1015, 2011.

[19] B. Babadi and H. Sompolinsky, "Sparseness and expansion in sensory representations," *Neuron*, vol. 83, no. 5, pp. 1213–1226, 2014.

[20] S. X. Luo, R. Axel, and L. Abbott, "Generating sparse and selective third-order responses in the olfactory system of the fly," *Proceedings of the National Academy of Sciences*, vol. 107, no. 23, pp. 10713–10718, 2010.

[21] A. Litwin-Kumar, K. D. Harris, R. Axel, H. Sompolinsky, and L. Abbott, "Optimal degrees of synaptic connectivity," *Neuron*, vol. 93, no. 5, pp. 1153–1164, 2017.

[22] L. B. Haberly, "Parallel-distributed processing in olfactory cortex: new insights from morphological and physiological analysis of neuronal circuitry," *Chemical senses*, vol. 26, no. 5, pp. 551–576, 2001.

[23] S. Dasgupta, C. F. Stevens, and S. Navlakha, "A neural algorithm for a fundamental computing problem," *Science*, vol. 358, no. 6364, pp. 793–796, 2017.

[24] K. I. Nagel and R. I. Wilson, "Biophysical mechanisms underlying olfactory receptor neuron dynamics," *Nature neuroscience*, vol. 14, no. 2, pp. 208–216, 2011.

[25] P. Sanda, T. Kee, N. Gupta, M. Stopfer, and M. Bazhenov, "Classification of odorants across layers in locust olfactory pathway," *Journal of neurophysiology*, vol. 115, no. 5, pp. 2303–2316, 2016.

[26] M. Rabinovich, R. Huerta, A. Volkovskii, H. Abarbanel, M. Stopfer, and G. Laurent, "Dynamical coding of sensory information with competitive networks," *Journal of Physiology-Paris*, vol. 94, no. 5-6, pp. 465–471, 2000.

[27] O. Mazor and G. Laurent, "Transient dynamics versus fixed points in odor representations by locust antennal lobe projection neurons," *Neuron*, vol. 48, no. 4, pp. 661–673, 2005.

[28] G. C. Turner, M. Bazhenov, and G. Laurent, "Olfactory representations by drosophila mushroom body neurons," *Journal of neurophysiology*, vol. 99, no. 2, pp. 734–746, 2008.

[29] G. Laurent, "Olfactory network dynamics and the coding of multidimensional signals," *Nature reviews neuroscience*, vol. 3, no. 11, pp. 884–895, 2002.

[30] N. Gupta and M. Stopfer, "A temporal channel for information in sparse sensory coding," *Current Biology*, vol. 24, no. 19, pp. 2247–2256, 2014.

[31] S. L. Brown, J. Joseph, and M. Stopfer, "Encoding a temporally structured stimulus with a temporally structured neural representation," *Nature neuroscience*, vol. 8, no. 11, pp. 1568–1576, 2005.

[32] B. Raman, J. Joseph, J. Tang, and M. Stopfer, "Temporally diverse firing patterns in olfactory receptor neurons underlie spatiotemporal neural codes for odors," *Journal of Neuroscience*, vol. 30, no. 6, pp. 1994–2006, 2010.

[33] A. Grabska-Barwińska, S. Barthelmé, J. Beck, Z. F. Mainen, A. Pouget, and P. E. Latham, "A probabilistic approach to demixing odors," *Nature neuroscience*, vol. 20, no. 1, pp. 98–106, 2017.

[34] N. Hiratani and P. E. Latham, "Rapid bayesian learning in the mammalian olfactory system," *Nature communications*, vol. 11, no. 1, pp. 1–15, 2020.

[35] B. A. Olshausen and D. J. Field, "Emergence of simple-cell receptive field properties by learning a sparse code for natural images," *Nature*, vol. 381, no. 6583, pp. 607–609, 1996.

[36] D. H. Hubel and T. N. Wiesel, "Receptive fields, binocular interaction and functional architecture in the cat's visual cortex," *The Journal of physiology*, vol. 160, no. 1, p. 106, 1962.

[37] M. Riesenhuber and T. Poggio, "Models of object recognition," *Nature neuroscience*, vol. 3, no. 11, pp. 1199–1204, 2000.

[38] K. Krishnamurthy, A. M. Hermundstad, T. Mora, A. Walczak, C. F. Stevens, and V. Balasubramanian, "The functional role of randomness in olfactory processing.," *COSYNE Abstracts 2014, Salt Lake City*, 2014.

[39] C. W. Yu, K. A. Prokop-Prigge, L. A. Warrenburg, and J. D. Mainland, "Drawing the borders of olfactory space," in *Chemical Senses*, vol. 40, pp. 565–565, OXFORD UNIV PRESS GREAT CLARENDON ST, OXFORD OX2 6DP, ENGLAND, 2015.

[40] R. G. Baraniuk, V. Cevher, and M. B. Wakin, "Low-dimensional models for dimensionality reduction and signal recovery: A geometric perspective," *Proceedings of the IEEE*, vol. 98, no. 6, pp. 959–971, 2010.

[41] D. L. Donoho, "Compressed sensing," *IEEE Transactions on information theory*, vol. 52, no. 4, pp. 1289–1306, 2006.

[42] E. J. Candès, J. Romberg, and T. Tao, "Robust uncertainty principles: Exact signal reconstruction from highly incomplete frequency information," *IEEE Transactions on information theory*, vol. 52, no. 2, pp. 489–509, 2006.

[43] T. M. Cover, "Geometrical and statistical properties of systems of linear inequalities with applications in pattern recognition," *IEEE transactions on electronic computers*, no. 3, pp. 326–334, 1965.

[44] L. Buck and R. Axel, "A novel multigene family may encode odorant receptors: a molecular basis for odor recognition," *Cell*, vol. 65, no. 1, pp. 175–187, 1991.

[45] E. A. Hallem and J. R. Carlson, "Coding of odors by a receptor repertoire," *Cell*, vol. 125, no. 1, pp. 143–160, 2006.

[46] A. F. Carey, G. Wang, C.-Y. Su, L. J. Zwiebel, and J. R. Carlson, "Odorant reception in the malaria mosquito anopheles gambiae," *Nature*, vol. 464, no. 7285, pp. 66–71, 2010.

[47] R. Tabor, E. Yaksi, J.-M. Weislogel, and R. W. Friedrich, "Processing of odor mixtures in the zebrafish olfactory bulb," *Journal of Neuroscience*, vol. 24, no. 29, pp. 6611–6620, 2004.

[48] M. L. Fletcher, "Analytical processing of binary mixture information by olfactory bulb glomeruli," *PLoS One*, vol. 6, no. 12, p. e29360, 2011.

[49] K. J. Grossman, A. K. Mallik, J. Ross, L. M. Kay, and N. P. Issa, "Glomerular activation patterns and the perception of odor mixtures," *European Journal of Neuroscience*, vol. 27, no. 10, pp. 2676–2685, 2008.

[50] D. Rokni, V. Hemmelder, V. Kapoor, and V. N. Murthy, "An olfactory cocktail party: figure-ground segregation of odorants in rodents," *Nature neuroscience*, vol. 17, no. 9, pp. 1225–1232, 2014.

[51] V. Singh, N. R. Murphy, V. Balasubramanian, and J. D. Mainland, "Competitive binding predicts nonlinear responses of olfactory receptors to complex mixtures," *Proceedings of the National Academy of Sciences*, vol. 116, no. 19, pp. 9598–9603, 2019.

[52] V. Singh, M. Tchernookov, and V. Balasubramanian, "What the odor is not: Estimation by elimination," *Physical Review E*, vol. 104, no. 2, p. 024415, 2021.

[53] G. Reddy, J. D. Zak, M. Vergassola, and V. N. Murthy, "Antagonism in olfactory receptor neurons and its implications for the perception of odor mixtures," *Elife*, vol. 7, p. e34958, 2018.

[54] J. D. Zak, G. Reddy, M. Vergassola, and V. N. Murthy, "Antagonistic odor interactions in olfactory sensory neurons are widespread in freely breathing mice," *Nature communications*, vol. 11, no. 1, pp. 1–12, 2020.

[55] E. J. Candès and Y. Plan, "Near-ideal model selection by l_1 minimization," *The Annals of Statistics*, vol. 37, no. 5A, pp. 2145–2177, 2009.

[56] C. J. Rozell, D. H. Johnson, R. G. Baraniuk, and B. A. Olshausen, "Sparse coding via thresholding and local competition in neural circuits," *Neural computation*, vol. 20, no. 10, pp. 2526–2563, 2008.

[57] S. R. Olsen, V. Bhandawat, and R. I. Wilson, "Divisive normalization in olfactory population codes," *Neuron*, vol. 66, no. 2, pp. 287–299, 2010.

[58] S. R. Olsen and R. I. Wilson, "Lateral presynaptic inhibition mediates gain control in an olfactory circuit," *Nature*, vol. 452, no. 7190, pp. 956–960, 2008.

[59] M. T. Wiechert, B. Judkewitz, H. Riecke, and R. W. Friedrich, "Mechanisms of pattern decorrelation by recurrent neuronal circuits," *Nature neuroscience*, vol. 13, no. 8, pp. 1003–1010, 2010.

[60] S. E. McGuire, P. T. Le, and R. L. Davis, "The role of drosophila mushroom body signaling in olfactory memory," *Science*, vol. 293, no. 5533, pp. 1330–1333, 2001.

[61] M. Heisenberg, A. Borst, S. Wagner, and D. Byers, "Drosophila mushroom body mutants are deficient in olfactory learning," *Journal of neurogenetics*, vol. 2, no. 1, pp. 1–30, 1985.

[62] S. J. Caron, V. Ruta, L. Abbott, and R. Axel, "Random convergence of olfactory inputs in the drosophila mushroom body," *Nature*, vol. 497, no. 7447, pp. 113–117, 2013.

[63] D. L. Sosulski, M. L. Bloom, T. Cutforth, R. Axel, and S. R. Datta, "Distinct representations of olfactory information in different cortical centres," *Nature*, vol. 472, no. 7342, pp. 213–216, 2011.

[64] D. D. Stettler and R. Axel, "Representations of odor in the piriform cortex," *Neuron*, vol. 63, no. 6, pp. 854–864, 2009.

[65] O. Barak, M. Rigotti, and S. Fusi, "The sparseness of mixed selectivity neurons controls the generalization–discrimination trade-off," *Journal of Neuroscience*, vol. 33, no. 9, pp. 3844–3856, 2013.

[66] D. Zwicker, A. Murugan, and M. P. Brenner, "Receptor arrays optimized for natural odor statistics," *Proceedings of the National Academy of Sciences*, vol. 113, no. 20, pp. 5570–5575, 2016.

[67] A. Mayer, V. Balasubramanian, T. Mora, and A. M. Walczak, "How a well-adapted immune system is organized," *Proceedings of the National Academy of Sciences*, vol. 112, no. 19, pp. 5950–5955, 2015.

[68] V. Venturi, D. A. Price, D. C. Douek, and M. P. Davenport, "The molecular basis for public t-cell responses?," *Nature Reviews Immunology*, vol. 8, no. 3, pp. 231–238, 2008.

[69] N. Thomas, K. Best, M. Cinelli, S. Reich-Zeliger, H. Gal, E. Shifrut, A. Madi, N. Friedman, J. Shawe-Taylor, and B. Chain, "Tracking global changes induced in the cd4 t-cell receptor repertoire by immunization with a complex antigen using short stretches of cdr3 protein sequence,"

Bioinformatics, vol. 30, no. 22, pp. 3181–3188, 2014.

[70] Y. Elhanati, A. Murugan, C. G. Callan, T. Mora, and A. M. Walczak, "Quantifying selection in immune receptor repertoires," *Proceedings of the National Academy of Sciences*, vol. 111, no. 27, pp. 9875–9880, 2014.

[71] D. R. Kepple, H. Giaffar, D. Rinberg, and A. A. Koulakov, "Deconstructing odorant identity via primacy in dual networks," *Neural computation*, vol. 31, no. 4, pp. 710–737, 2019.

[72] E. Gruntman and G. C. Turner, "Integration of the olfactory code across dendritic claws of single mushroom body neurons," *Nature neuroscience*, vol. 16, no. 12, pp. 1821–1829, 2013.

[73] C. Schroll, T. Riemensperger, D. Bucher, J. Ehmer, T. Völler, K. Erbguth, B. Gerber, T. Hendel, G. Nagel, E. Buchner, *et al.*, "Light-induced activation of distinct modulatory neurons triggers appetitive or aversive learning in drosophila larvae," *Current biology*, vol. 16, no. 17, pp. 1741–1747, 2006.

[74] A. Fiala, "Olfaction and olfactory learning in drosophila: recent progress," *Current opinion in neurobiology*, vol. 17, no. 6, pp. 720–726, 2007.

[75] M. Rigotti, O. Barak, M. R. Warden, X.-J. Wang, N. D. Daw, E. K. Miller, and S. Fusi, "The importance of mixed selectivity in complex cognitive tasks," *Nature*, vol. 497, no. 7451, pp. 585–590, 2013.

[76] C. D. Wilson, G. O. Serrano, A. A. Koulakov, and D. Rinberg, "A primacy code for odor identity," *Nature communications*, vol. 8, no. 1, pp. 1–10, 2017.

[77] A. Dewan, A. Cichy, J. Zhang, K. Miguel, P. Feinstein, D. Rinberg, and T. Bozza, "Single olfactory receptors set odor detection thresholds," *Nature communications*, vol. 9, no. 1, pp. 1–12, 2018.

[78] G. Tavoni, D. Kersen, and V. Balasubramanian, "Cortical feedback and gating in odor discrimination and generalization," *PLoS Computational Biology*, vol. 17, no. 19, p. e1009479, 2021.

[79] D. Kersen, G. Tavoni, and V. Balasubramanian, "Connectivity and dynamics in the olfactory bulb," *PLoS Computational Biology*, vol. 18, no. 2, p. e1009856, 2022.

[80] R. Chartrand and W. Yin, "Iteratively reweighted algorithms for compressive sensing," in *2008 IEEE international conference on acoustics, speech and signal processing*, pp. 3869–3872, IEEE, 2008.

[81] E. J. Candes and T. Tao, "Near-optimal signal recovery from random projections: Universal encoding strategies?," *IEEE transactions on information theory*, vol. 52, no. 12, pp. 5406–5425, 2006.

[82] F. Pedregosa, G. Varoquaux, A. Gramfort, V. Michel, B. Thirion, O. Grisel, M. Blondel, P. Prettenhofer, R. Weiss, V. Dubourg, *et al.*, "Scikit-learn: Machine learning in python," *the Journal of machine Learning research*, vol. 12, pp. 2825–2830, 2011.
